# Intercellular communication is required for trap formation in the nematode-trapping fungus *Duddingtonia flagrans*

**DOI:** 10.1371/journal.pgen.1008029

**Published:** 2019-03-27

**Authors:** Loubna Youssar, Valentin Wernet, Nicole Hensel, Xi Yu, Heinz-Georg Hildebrand, Birgit Schreckenberger, Marius Kriegler, Birgit Hetzer, Phillip Frankino, Andrew Dillin, Reinhard Fischer

**Affiliations:** 1 Department of Microbiology, Karlsruhe Institute of Technology (KIT)—South Campus, Institute for Applied Biosciences, Karlsruhe, Germany; 2 Max Rubner Institute, Karlsruhe, Germany; 3 Department of Molecular and Cell Biology, Howard Hughes Medical Institute, University of Berkeley, Berkeley, California, United States of America; Oregon State University, UNITED STATES

## Abstract

Nematode-trapping fungi (NTF) are a large and diverse group of fungi, which may switch from a saprotrophic to a predatory lifestyle if nematodes are present. Different fungi have developed different trapping devices, ranging from adhesive cells to constricting rings. After trapping, fungal hyphae penetrate the worm, secrete lytic enzymes and form a hyphal network inside the body. We sequenced the genome of *Duddingtonia flagrans*, a biotechnologically important NTF used to control nematode populations in fields. The 36.64 Mb genome encodes 9,927 putative proteins, among which are more than 638 predicted secreted proteins. Most secreted proteins are lytic enzymes, but more than 200 were classified as small secreted proteins (< 300 amino acids). 117 putative effector proteins were predicted, suggesting interkingdom communication during the colonization. As a first step to analyze the function of such proteins or other phenomena at the molecular level, we developed a transformation system, established the fluorescent proteins GFP and mCherry, adapted an assay to monitor protein secretion, and established gene-deletion protocols using homologous recombination or CRISPR/Cas9. One putative virulence effector protein, PefB, was transcriptionally induced during the interaction. We show that the mature protein is able to be imported into nuclei in *Caenorhabditis elegans* cells. In addition, we studied trap formation and show that cell-to-cell communication is required for ring closure. The availability of the genome sequence and the establishment of many molecular tools will open new avenues to studying this biotechnologically relevant nematode-trapping fungus.

## Author summary

Nematode-trapping fungi are fascinating microorganisms, because they are able to switch from saprotrophic growth to a predatory lifestyle. *Duddingtonia flagrans* forms adhesive trap systems and conidia and resistant chlamydospores. Chlamydospores are ideal for dissemination in the environment to control nematode populations in the field. We show that *D*. *flagrans* is able to catch *C. elegans* but also the very large wine-pathogenic nematode *Xiphinema index*. We sequenced the *D*. *flagrans* genome and show that it encodes about 10,000 genes with a large proportion of secreted proteins. We hypothesize that virulence effector proteins are involved in the interkingdom organismic interaction and identified more than 100 candidates. In order to investigate the molecular biology of *D*. *flagrans* and its interaction with nematodes, we established a transformation system and several molecular tools. We show that cell-to-cell communication and hyphal fusion are required for trap formation. Finally, we show that one putative virulence effector protein targets nuclei when expressed in *C*. *elegans*.

## Introduction

A predatory lifestyle is typically associated with animals like lions, tigers or snakes. Dramatic hunts or attacks out of a hiding place are typical scenes in reportage movies. No less dramatic is the situation when some nematode-trapping fungi (NTF) catch their prey using sophisticated trapping systems. NTF are an important group of soil microorganisms, which are able to live also saprotrophically. Some other nematode-attacking fungi are obligate pathogens [1–3]. NTF have evolved a variety of trapping structures, including constricting rings and five types of adhesive traps (sessile adhesive knobs, stalked adhesive knobs, adhesive nets, adhesive columns, and non-constricting rings) with which they capture nematodes to supplement their diet. There is evidence that NTF originated in the Permian and Triassic [4]. One hypothesis is that dead creatures caused by mass extinctions at that time were rich in carbon but poor in nitrogen and hence direct capture of nitrogen-rich living animals would give predatory fungi a competitive advantage over strictly saprotrophic fungi. Adhesive, non-constricting rings or hyphal loops are considered as ancient structures, after which constricting rings evolved [4].

The organismic interaction between NTF and nematodes reflects more than 400 million years of co-evolution and is highly sophisticated [4]. NTF “smell” their prey and only form traps in the presence of nematodes [5]. On the other hand, fungi secrete volatile substances to attract the worms [6]. It was hypothesized that chemical warfare and sophisticated communication between the two partners occurs also after penetration of the hyphae into the nematode [7]. It is conceivable that secreted proteins may fulfill such functions as described in plant-fungal interactions. For instance, the oomycete Phytophthora infestans attenuates the plant defense system with secreted effector proteins before killing the host cells [8]. In *U. maydis* the effector Rsp3 is exposed at the hyphal surface during biotrophic growth and inactivates two antifungal peptides [9]. Likewise, the arbuscular mycorrhizal fungus *Glomus intraradices* secretes an effector that suppresses the host defense and thereby contributes to the establishment of a long-term symbiotic relationship [10]. Hence, effectors play key roles in modulating host cell physiology to promote virulence, biotrophic growth or symbiosis [11–14]. In NTF, certain secreted proteins may be used for modulating the innate immune system of the nematode or target other intracellular processes. Such fungal proteins would share a secretion signal and may contain additional signals for subcellular localization within the host cells. These motifs could be organellar targeting signals, such as the NLS found in the *U. maydis* effector, See1 (seedling efficient effector 1), which reactivates host DNA synthesis and cell division [15].

In addition, to the fascinating predatory lifestyle of NTF, NTF were applied as biocontrol agents against nematodes, which colonize the gastrointestinal tract of sheep. Heavy loads of nematodes may cause dramatic reductions in productivity [16]. As nematode eggs are secreted with the feces, and sheep are grazing around their feces, re-infections are very common and thus antihelmintic treatments are only of temporary help. Excitingly, NTF can be used to reduce the nematode number in a field. *Duddingtonia flagrans* and *Monacrosporium thaumasium* have the greatest potential as biocontrol agent because they form resistant chlamydospores [17, 18]. It has been shown that chlamydospores are able to pass the gastrointestinal tract of animals and germinate in the feces where they reduced the number of nematodes [19, 20]. Multinutritional pellets containing *D*. *flagrans* chlamydospores were already used in sheep and horses [21–23].

Nematodes are also a threat for many crop plants. For instance, the nematode *Heterodera schachtii* attacks sugar beet plants and may cause complete loss of the harvest [24]. There are no effective treatments of such pests, besides cultivation plans with different crops. At least six years are required until the reservoir of infective nematodes is cured. Another example is *Xiphinema index*, a very large pathogenic nematode, which attacks the roots of grapevine. Besides direct damage of the roots, *X. index* transmits the Grapevine fanleaf virus (GFLV), which causes large losses in the wine industry [25]. There are estimates that almost 10% of all Chardonnay Vineyards in France are infected with the virus. The last example is the attack of coffee plants by nematodes of the genera Helicotylenchus and Xiphinema [26]. There are estimates that nematodes cause annual losses of more than $ 80 billion worldwide [27].

In this paper, we studied *D*. *flagrans*, sequenced its DNA and annotated its genome, developed a transformation system with three different selection markers, established the use of the CRISPR/Cas9 technology and fluorescent proteins for subcellular localization. With these tools, we studied a putative fungal virulence factor, PefB. We show that it is a secreted protein that is upregulated during infection. The mature protein was expressed in *C. elegans* where it is localized to nuclei. Finally, we generated hypotheses to analyze chlamydospore morphogenesis and proved the hypothesis that intercellular communication is required for ring closure during trap formation.

## Results and discussion

### *D*. *flagrans* forms chlamydospores for survival and adhesive traps to catch nematodes

**Fig 1:**
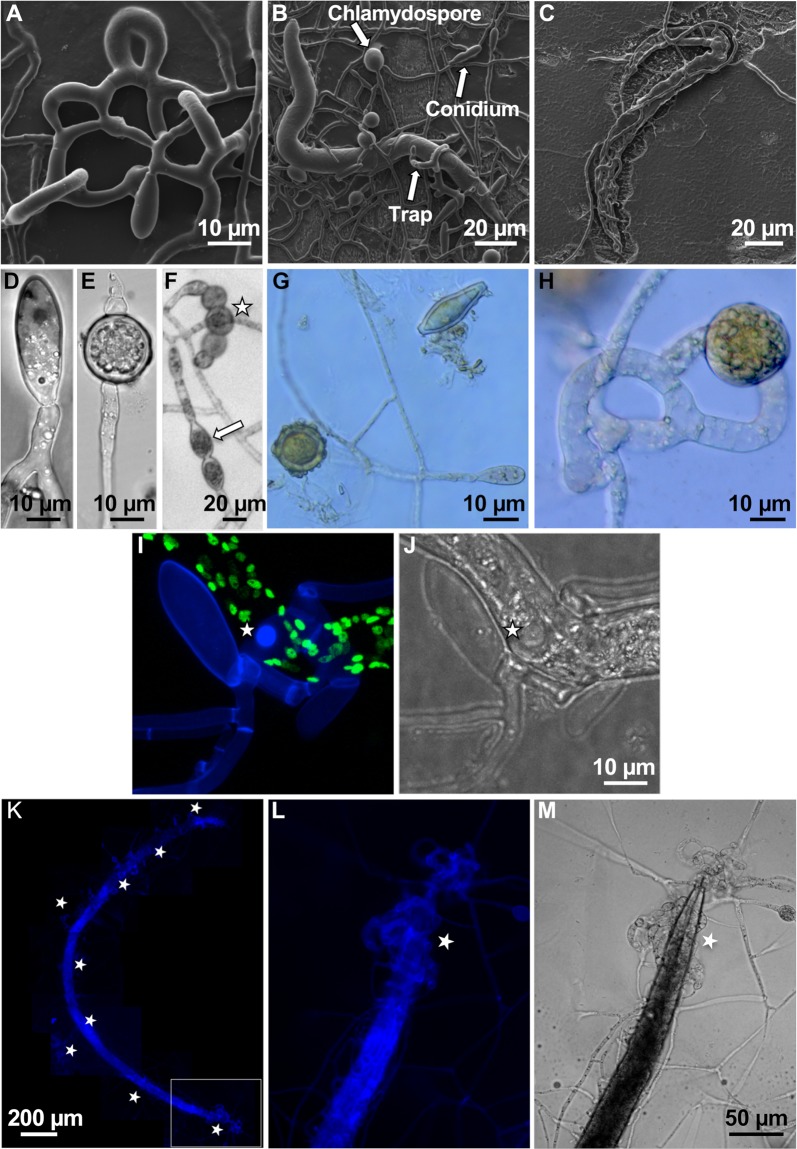
The nematode-trapping fungus *D*. *flagrans* produces adhesive traps, spores, and chlamydospores. **(A)** Formation of a three-dimensional trapping network. **(B)** Trapped nematode *C. elegans*. Chlamydospores, normal spores and traps are labelled with arrows. **(C)** Complete degradation of *C. elegans* and fungal growth inside the nematode. **(D)** Asexual spore. **(E)** Chlamydospore. **(F)** Conversion of single trap-compartments into chlamydospores (star) compared to the conversion of vegetative cells (arrow). **(G, H)** Glycogen staining with Lugol‘s iodine. **(I, J)** Visualization of ring-like accumulation of chitin (star) at the contact zone of trap cells with the nematode cuticle. The cell wall of the fungus was stained with calcofluor-white (CFW). Nuclei of C. elegans were labelled with GFP [78]. **(K)** A *X. index* adult trapped in multiple *D*. *flagrans* networks (stars) after 48 h of co-incubation. The nematode body is completely filled with hyphae, revealed by CFW staining. **(L, M)** Magnification of the framed area in **(I)** and turned 90 degrees counter clockwise. The star indicates the trapping network around the head region of the nematode.

It is very favorable for the application of microorganisms as biocontrol agents if (1) the organisms are easy to cultivate and if (2) robust cellular structures are produced, which can be used to disseminate the agent; both apply to *D*. *flagrans*. In comparison to other NTF, *D*. *flagrans* grows relatively fast under laboratory conditions on typical media on agar plates. Two days after point inoculation on a potato-dextrose-agar (PDA) plate a colony of 1 cm diameter was observed. *D*. *flagrans* is able to capture nematodes with three-dimensional trapping networks. In order to induce trap formation, we inoculated *D*. *flagrans* spores on low nutrient agar (LNA) and added around 100 individuals of *C*. *elegans* to the plate. After 6 h incubation at room temperature, the first forming traps were observed. After 12 h, the first trap networks were complete, and nematodes trapped. Completely digested nematodes were usually found after 24 h **(Fig 1A–1C)**.

*D*. *flagrans* produces ovoid-shaped asexual spores (conidia) at the tip of conidiophores after 24 h **(Fig 1D)**, and, under starvation conditions, vegetative hyphae are able to differentiate into asexual spores resistant for survival (chlamydospores)**(Fig 1E)** [18]. Under laboratory conditions, the formation of chlamydospores was observed after 3 days of growth on 6 cm LNA Petri dishes and incubation at 28°C. Usually chlamydospores develop from vegetative compartments or at hyphal tips. In addition, we observed that after at least 48 h of co-incubation with *C. elegans* single compartments of empty traps transformed into chlamydospores **(Fig 1F)**. In order to show that the round spores and the transformed trap compartments were indeed chlamydospores, we tested if they contain glycogen as storage material. When incubated with Lugol’s iodide, chlamydospores showed a brown color, characteristic for glycogen staining, whereas hyphae and conidia were much less or not stained (**Fig 1G and 1H**).

To analyze the predation process in detail, fluorescent microscopy was used. In order to visualize fungal hyphae inside the nematode, fungal cell walls were stained with calcofluor white **(Fig 1I)**, and *C. elegans* nuclei were visualized by GFP expression. During early stages of the interaction, we observed a ring-like accumulation of chitin at the contact zone of *D*. *flagrans* trap cells with the nematode cuticle **(Fig 1I and 1J)**. These accumulations likely represent penetration tubes emerging at the anchorage zone 2–4 h after adhesion [3, 28].

To test if *D*. *flagrans* is able to trap and inactivate plant-pathogenic nematodes, the grapevine-pathogenic nematode *X. index* was tested as prey. X. index is an obligate pathogen and can be cultivated on grapevine plants or fig trees [29]. X. index is much larger than *C. elegans*. Adults, which are 3 mm in length, were hand-picked under a stereo microscope and in total 10 individuals were added to 5x10^4^ *D*. *flagrans* spores on LNA plates. After 12 h co-incubation first traps were observed and after 24 h first immobilized nematodes. After 48 h the first nematodes were digested **(Fig 1K–1M)**. These results show that *D*. *flagrans* is able to attack different nematodes and has thus the potential to be developed as a biocontrol agent against plant-pathogenic nematodes.

### The genome of *D*. *flagrans* and comparison with other NTF

*D*. *flagrans* genomic DNA was sequenced using Pacific Biosciences SMRT to generate 411,097 reads with an average read length of 8,516 nt. The genome was assembled de novo and 88% of reads were mapped against the genome of the related NTF *Arthrobotrys oligospora*. The average depth coverage was 102.18 **(S1 Table)**. The high-quality sequence was assembled into 9 contigs, reaching from 8,347,096 bp to 28,584 bp in size **(Table 1, S1 Table)**, summing up to 36.64 Mb and with an overall G+C content of 45.5% **(S1 Fig, S2 Table)**. Six contigs had sizes between 4.4 Mb and 8 Mb, whereas the other eight contigs were only between 0.28 Mb and 0.028 Mb in length. The six large contigs may represent six chromosomes.

**Table 1:** Features of the *D*. *flagrans* genome.

Sequencing Features	Value
Contigs	14
Number of reads	411,097
N50 Read Length	17,206
Number of contigs	14
Max Contig Length	8,347,096
N50 Contig Length	6,168,687
N50 length of scaffolds (bp)	3/6.169
N90 length of scaffolds (bp)	6/4.095
Number of scaffolds > 50 KB	9
% main genome in scaffolds > 50 KB	99.50%
Genome size (Mb)	36.64
(G + C) percentage	45.1
Total length of coding sequences (Mb)	1.44
Repeat content (percentage)	2.18
tRNA genes	159
Average gene size (kb)	1.49
Average number of exons per gene	3.80
Average number of introns per gene	2.75
Average CDS size (min; max)	485.6 codons (202; 36,201)
Average intron length (bp)	92
Number of protein-encoding genes	9927
Secretome	638 (~6.4%)

In order to improve the annotation, we performed an RNAseq analysis. RNA was extracted from *D*. *flagrans* saprotrophic and zootrophic stages and pooled to one sample. The RNA of the different developmental stages should increase the possibility that a maximum number of genes would be expressed and detected in the RNAseq analysis. The sample was sequenced using the Ilumina platform (BGISEQ-500) at the Beijing Genomics Institute (Beijing, China). 23,941,723 reads were generated and 91.79% were mapped to the *D*. *flagrans* genome. 88.84% had a unique match. Structural gene annotation was then done with BRAKER2. 9,927 genes were predicted, which is close to the gene number in *A*. *oligospora* and similar to the coding capacity of other ascomycetes **(S3 Table)**. Among the 9,927 protein-coding genes only 5978 showed homology to proteins deposited in the Swiss-Prot database **(S4 Table)**. These results indicate the presence of a high number of novel proteins in *D*. *flagrans*. Using *A*. *oligospora* as database, we found 9,336 similar proteins in *D*. *flagrans* **(S5 Table)**. Subcellular localization predictions revealed 41% nucleic, 23% cytosolic, 19% mitochondrial and 12% extracellular proteins. Mitochondrial genes were also annotated **(S6 Table)**.

**Fig 2:**
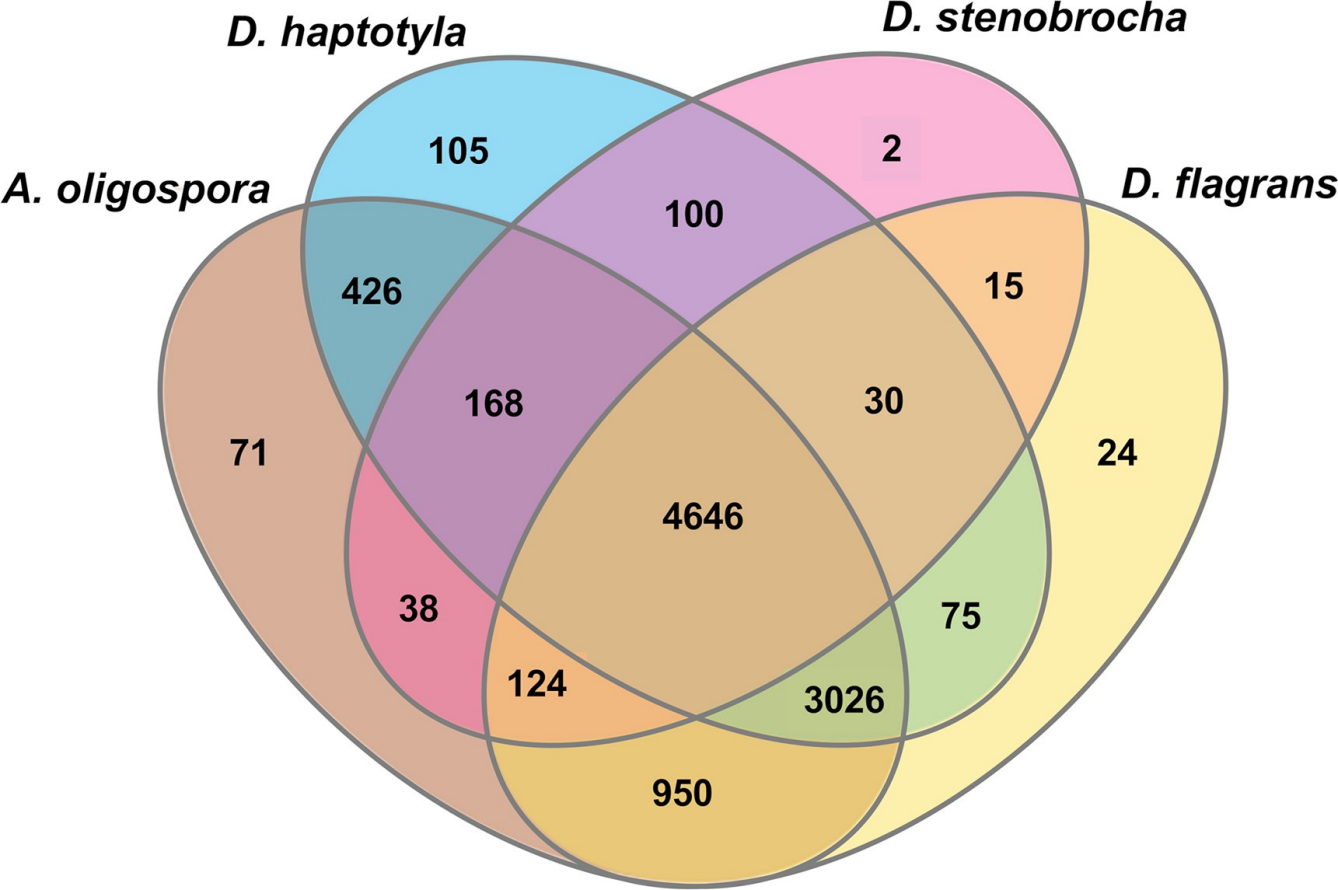
Orthologous clusters in the proteomes of four nematode-trapping fungi. A Venn diagram plotted by OrthoVenn shows shared orthologous protein clusters among *D*. *flagrans*, *A*. *oligospora*, *Da*. *haptotyla*, and *Dr*. *stenobrocha* proteomes. Most gene families were shared between the closely related *A*. *oligospora* and *D*. *flagrans*. All four nematode-trapping fungi shared 4646 orthologous genes clusters.

To annotate orthologous proteins, the proteome of *D*. *flagrans* was compared (BLASTP) to the proteomes of other NTF using a threshold of 80% predicted amino-acid similarity with at least 50% coverage. We found 7,488, 4,373 and 2,352 orthologous proteins in *A. oligospora*, Dactylellina haptotyla (abbreviated as Da. haptotyla), and Drechslerella stenobrocha (abbreviated as *Dr. stenobrocha*), respectively. The four species were used to generate a Venn diagram of orthologous clusters **(Fig 2)**. Orthologous proteins gathered together in the same cluster, and among the total number of 9,800 clusters, 9,598 were orthologues (at least present in two species) and 4,475 were single-copy gene clusters **(Table 2)**. 4,646 cluster gene families were orthologous in all four fungi. Moreover, *D*. *flagrans* shares 950, 75 and 15 gene families with *A*. *oligospora*, *Da*. *haptotyla* and *Dr*. *stenobrocha*, respectively. The highest number of clusters shared between *D*. *flagrans* and *A*. *oligospora* reflects their close relationship **(Fig 2 and Table 2)**. 855 proteins of *D*. *flagrans* had no orthologous proteins with any of the other three species **(Table 2)**. We also analyzed the proteome for potential genes involved in the worm attack. We assume that such proteins may be related to proteins involved in pathogenic interactions. Therefore, we analyzed the *D*. *flagrans* proteome using the InterProScan and the Pathogen Host Interactions (PHI) databases **(S1 Text, S7–S10 Tables)**. Additionally, we analyzed the phylogenetic relationship between *D*. *flagrans* and 13 other filamentous fungi by using orthologous proteins **(S3 Fig)(S1 Text)**.

**Table 2:** Protein numbers, clusters and singletons generated from OrthoVenn when comparing *D*. *flagrans*, *A*. *oligospora*, *Da*. *haptotyla* and *Dr*. *stenobrocha*.

Species	Proteins	Clusters	Singletons
*D. flagrans*	9927	8890	855
*A. oligospora*	11479	9449	1723
*Da. haptotyla*	10959	8576	2017
*Dr. stenobrocha*	5597	5597	424

### Capacity for the production of secondary metabolites

Secondary metabolites are defined as metabolites which are not essential for the survival of the producing organism in the laboratory, many of which are only produced under specific growth or physiological conditions. A large proportion of secondary metabolites are polyketide or peptide derivatives, which are synthesized by polyketide synthases (PKS) and non-ribosomal peptide synthases (NRPS), respectively. The genome of *D*. *flagrans* harbors only three PKS and three NRPS type gene clusters [30]. In the genome of *A. oligospora*, five putative PKS and seven putative NRPS genes were found. Likewise, Drechslerella contains two putative PKS/PKS-NRPSs and three putative NRPSs [26]. These are rather low numbers as compared to other filamentous fungi. In *A. alternata* ten different PKS/PKS-NRPS and five NRPS clusters are found [31, 32], in *F. graminearum* 12 PKSs and 10 NRPSs [33], and in *A. flavus* even 29 PKSs and 27 NRPSs [34].

In *D*. *flagrans* one of the PKS displays similarity to 6-MSAS type polyketide synthases with strong homology to the PKS encoded by AOL_s00215g283. This PKS is described to be negatively involved in trap formation in *A*. *oligospora* [35]. It is likely that it fulfills a similar function in *D*. *flagrans*. The other 2 PKS genes (both type I PKS) could not yet be assigned a function, but they show high homology to the type I PKSs, AOL_s00215g926, AOL_s00079g496 and AOL_s00043g828, which were predicted to be involved in lovastatin biosynthesis [36].

### The secretome of *D*. *flagrans* and interkingdom communication

The fungal attack of nematodes requires a repertoire of lytic enzymes and perhaps other virulence factors or effectors to modulate the innate immune response or overcome the host defense. In order to identify candidates for both classes of proteins, we analyzed the putative secreted proteins in *D*. *flagrans*. Using SignalP, WolfPsort and TMHMM, we identified 638 proteins (~6.4% of the whole genome). Proteins with hydrolase activity, oxidoreductases and peptidase activity are the most abundant. Carbohydrate metabolic and other catabolic processes are also over-represented compared to other functions **(S11 Table)**.

**Fig 3:**
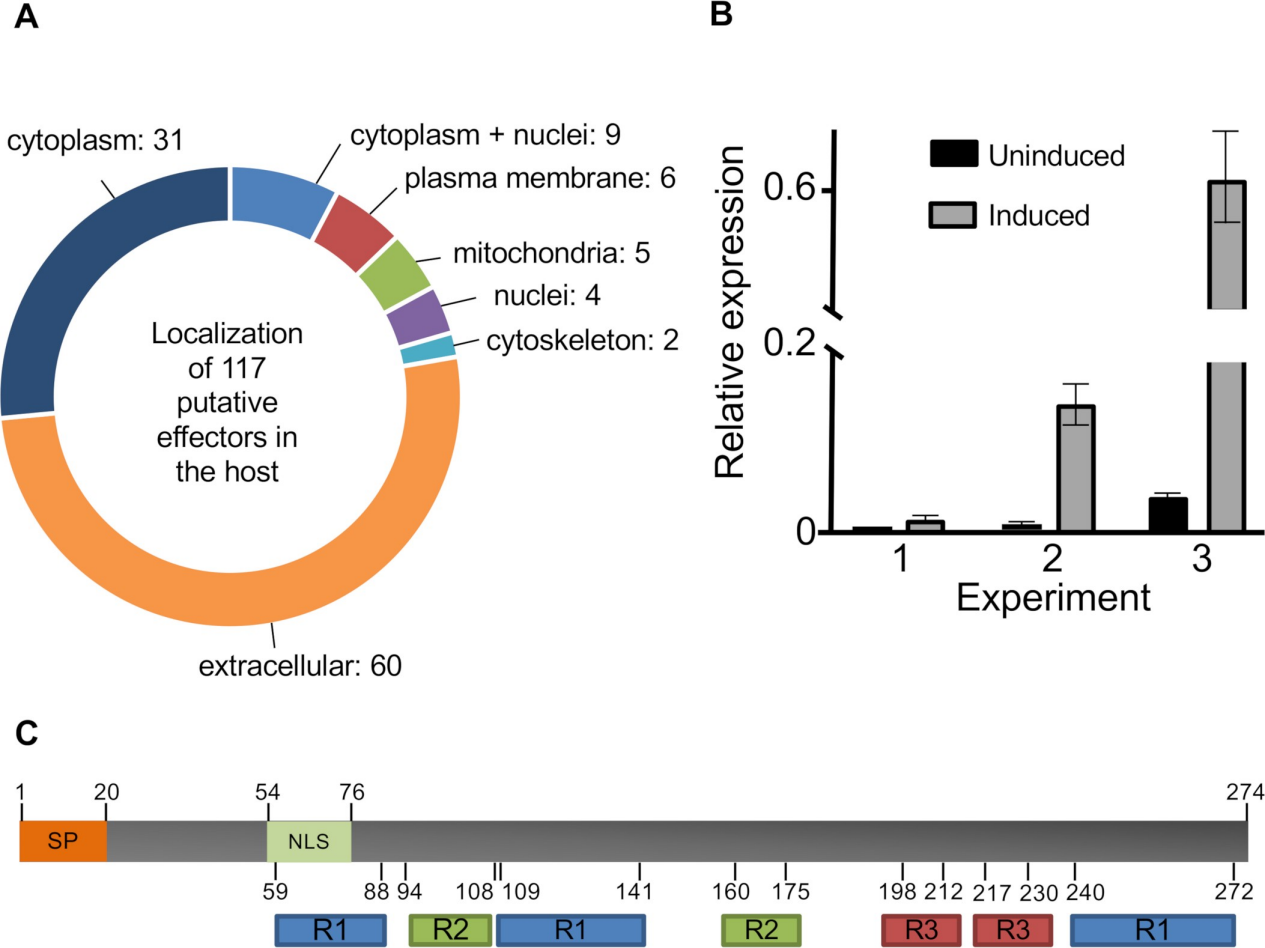
Predicted secreted proteins, SSPs and host localization of putative effectors. (A) 638 proteins without transmembrane domain (TMHMM) and with signal peptide (SignalP 4.0) were predicted to be secreted by WoLF PSORT. 249 of these proteins are SSPs (< 300 amino acids). 117 putative effector proteins or virulence factors were predicted with EffectorP 2.0. The sub-cellular localization of the mature proteins in the host was predicted using WoLF PSORT (animal mode). (B) Expression of *pefB* in hyphae of *D*. *flagrans* and in hyphae co-cultivated for 24 h with *C*. *elegans*. The expression was normalized to actin. (C) Scheme of PefB. The 274 amino acid (aa) protein comprises a 20 aa signal peptide at the N-terminus and an NLS between amino acids 54 and 76. Three repeat sequences (R1, 2, 3) were predicted by RADAR.

In order to identify putative effectors or virulence factors we applied the EffectorP 2.0 machine learning method for fungal effector prediction in secretomes. This tool is based on machine learning and is trained with fungal effectors as positive training set and secreted proteins not classified as effectors as the negative set [37]. 117 such proteins were found **(S12 Table)(Fig 3)**. We further used WoLF PSORT to predict the subcellular localization of the mature proteins (without secretion signal) to specific host cell organelles. Most proteins (60) were predicted to reside in the extracellular space. Here, a possible target could be the *C. elegans* cuticle collagen which plays an important role in barrier integrity and the immune response [38]. Nine putative effectors were predicted to enter nuclei.

Only 37 of the 117 proteins could be annotated. Three had predicted pectin lyase domains, four were annotated as glycoside hydrolase, 10 as other enzymes, two with domains of unknown function (DUF1524). A similar DUF1524 motif is found in protein families in the His-Me finger endonuclease superfamily, which suggests a function as endonuclease.

In another approach, the genome was analyzed for small secreted proteins (SSP) (< 300 amino acid). SSPs are involved in pathogenesis in numerous fungi [39] and the expression of many SSPs was shown to be highly upregulated in *A. oligospora* and *Da. haptotyla* during infection of the host. The *D*. *flagrans* secretome comprises 249 SSPs, compared to 312 in the facultative nematode endoparasite Dr. coniospora and 695 in the nematode-trapping fungus *Da*. *haptotyla* [40, 41]. 43 of the proteins identified with EffectorP were also identified as SSPs. Only 24 SSPs of *D*. *flagrans* had Pfam annotations in InterProScan. The lack of known homologs and Pfam domains of SSPs is consistent with observations in other fungi as *Da*. *haptotyla*, where rapid divergence of the SSPs was shown [42, 43]. 14 SSPs contained at least five cysteines probably stabilizing them. Using the PHI database 110 of the 249 SSPs where predicted effectors or virulence factors that are likely to act outside the fungal cell or inside the host cell. In the same way as described for *D*. *flagrans*, the secretomes of 21 other fungi were predicted **(S4A Fig, S12 Table, S1 Text)**.

### Chlamydospores make the difference

One major difference between *D*. *flagrans* and the closely related *A*. *oligospora* is the ability of *D*. *flagrans* to form resistant chlamydospores for survival. Hence, the genome of *D*. *flagrans* should contain a number of genes not present in *A*. *oligospora*. To generate a list of candidate proteins we compared the proteomes of *D*. *flagrans* and *A*. *oligospora*. 591 proteins were exclusive to *D*. *flagrans*. Only 189 proteins resulted in database hits (RefSeq and Swiss-Prot). A protein library of the 591 proteins was generated and compared to the proteomes of the four chlamydospore-forming fungi *Botrytis cinerea*, *Cryptococcus neoformans*, *Fusarium oxysporum* and *P*. *chlamydosporia* by protein database search (BLASTP) with 58, 29, 109 and 119 hits respectively. These proteins were then clustered using OrthoVenn **(S5 Fig)**. The 591 proteins from *D*. *flagrans* formed 81 clusters, including 8 orthologous clusters present in all five chlamydospore-forming fungi and correspond to 8 proteins in *D*. *flagrans*. One of the clusters did not have any Swiss-Prot hit or GO annotation, the others were annotated as uncharacterized protein YPR170W-B, lipase 2, cytochrome b-c1 complex subunit 9, protein transport protein GOT1, protein GET1, acyl-coenzyme A oxidase 2 and Dol-P-Man:Man(5)GlcNAc(2)-PP-Dol alpha-1,3-mannosyltransferase. Chlamydospores are enriched in glycogen (see above), and it was shown that in *Candida albicans* dolichyl-phosphate beta-D-mannosyltransferase 1 (Dpm1) and Dpm3 mutants are impaired in chlamydospore formation [44]. This could indicate a role of the *D*. *flagrans* Dol-P-Man:Man(5)GlcNAc(2)-PP-Dol alpha-1,3-mannosyltransferase DFL_006194 in chlamydospore formation.

### Development of a transformation system and establishment of fluorescent proteins

**Fig 4:**
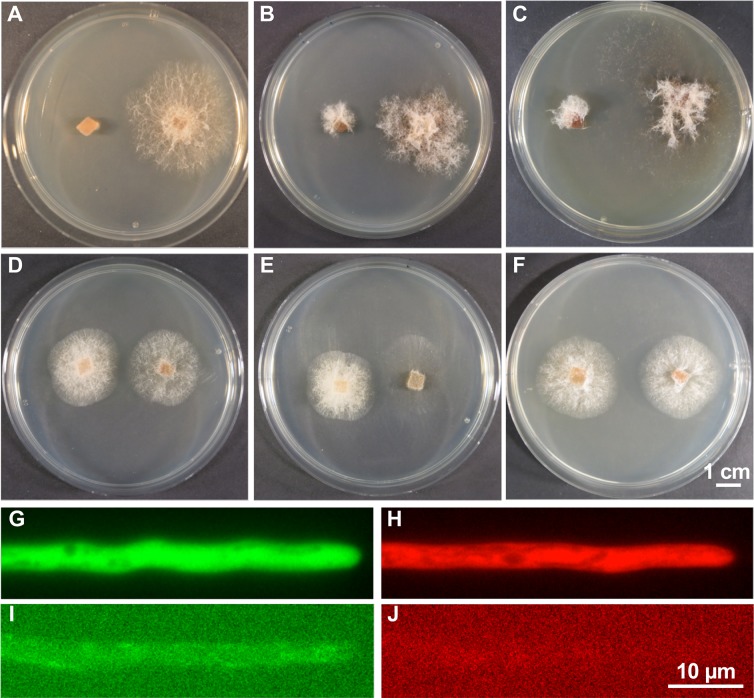
Establishment of a transformation system and fluorescent proteins in *D*. *flagrans*. **(A-C)** Comparison of the growth of *D*. *flagrans* wild type and transformants containing the hygromycin-B resistance gene *hph* **(A)**, the nourseothricin resistance gene *nat* **(B)**, or geneticin G418 resistance gene *neo* **(C)** on PDA supplemented with the corresponding drugs at inhibitory concentrations. Colonies were grown for 7 days at 28°C. **(D-F)** Comparison of the growth of *D*. *flagrans* wild type and corresponding transformants on PDA after 3 days incubation at 28°C. **(G)** Expression of GFP under the control of the *A*. *nidulans* *oliC* promoter. **(H)** Expression of mCherry under the control of the *A*. *nidulans* *oliC* promoter**. (I, J)** *D*. *flagrans* wild-type control with the same parameters as in **(G, H)**. Exposure time for **(G-J)** was 500 ms.

In order to further analyze the fungal-nematode interaction, genetic manipulations are required. As a first step, we adapted the protoplast preparation and transformation protocols from *A*. *oligospora* and *Aspergillus oryzae* for *D*. *flagrans* [45]. For this purpose, 24 h old *D*. *flagrans* liquid culture was used and prepared for protoplast formation and subsequent polyethylene glycol (PEG) / calcium chloride (CaCl_2_)–mediated DNA uptake. For selection of positive transformants the three dominant markers hygromycin-B, nourseothricin and geneticin (G418) were established. The drugs were efficient against protoplast regeneration and growth at 100 μg/ml hygromycin-B, 60 μg/ml nourseothricin or 120 μg/ml G418. To establish a transformation method, protoplasts were transformed with plasmids (pVW19, pRF62, pUMa1057) containing the hygromycin-B phosphotransferase gene *hph*, the nourseothricin acetyl transferase gene *nat* or the neomycin phosphotransferase gene *neo* [46]. Both the hph and nat resistance genes were under the control of the *A. nidulans trpC* promoter (ptrpC::hph and ptrpC::nat). The neo resistance gene was under the control of the *U. maydis o2tef* promoter (*po2tef::neo*). Mutants containing either of the resistance genes were able to grow on medium containing the corresponding drugs at concentrations where wild-type was inhibited **(Fig 4A–4F)**. The integration of the corresponding resistance genes into the genome was confirmed by PCR.

Next, we tested the expression of green (GFP) and red fluorescent proteins (mCherry). We tried corresponding genes, which were functional in *N. crassa*, in *A. nidulans*, in *U. maydis*, in *C. albicans* or in *Botrytis cinerea*. They were expressed using the A. nidulans glyceraldehyde-3-phosphate dehydrogenase (gpdA) promoter (*gpdA(p)::GFP* or *gpdA(p)::mCherry*). Only the version adapted for B. cinerea worked **(Fig 4G–4H)** [47]. Non-transformed wild-type hyphae did not show any green or red fluorescence **(Fig 4I–4J)**. This is the first report for the function of fluorescent proteins in NTF that form adhesive trapping networks.

**Fig 5:**
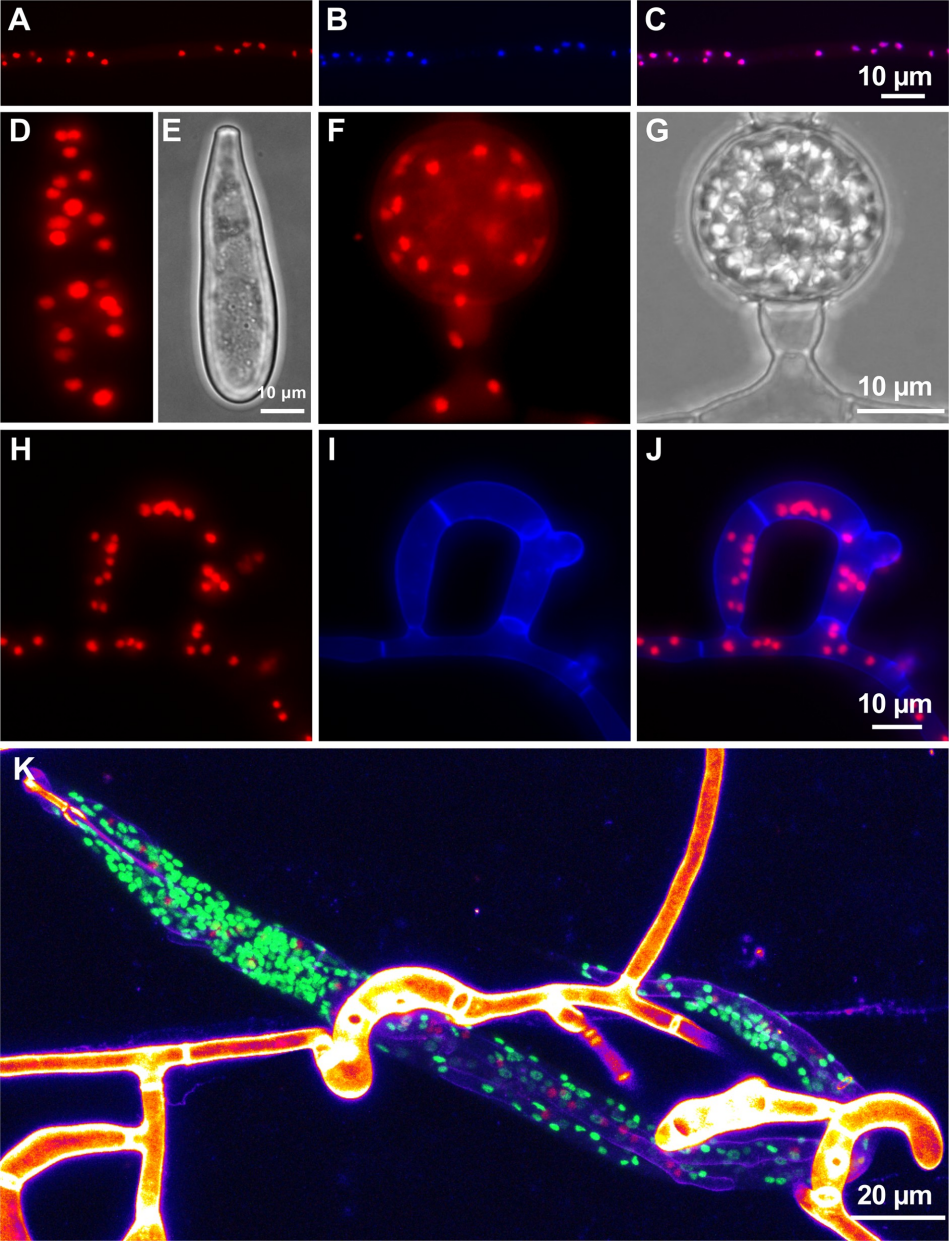
Visualization of *D*. *flagrans* nuclei using a H2B-mCherry fusion protein. H2B-mCherry was expressed under the *H2B* promoter in *D*. *flagrans*. (A-C) Hyphae. (B) Nuclei were visualized using Hoechst 33342, (C) Merge of (A) and (B). (D-G) Nuclei in a conidium and a chlamydospore. (H-J) Nuclei in a trap. (I) Calcofluor white (CFW) staining of the cell wall. (J) Merge of (H) and (I). (K) The fluorescent tag allowed to track the infection inside the nematode. Merged image of the GFP, mCherry and CFW channel. Nuclei of *C*. *elegans* were labelled with GFP. The cell wall of the fungus was stained using CFW. The contrast of the CFW channel was adjusted to see the weak staining of the fungal cell inside the nematode (purple) and is displayed as “Fire” using the ImageJ lookup table. The pixel values of the cell wall outside the nematode are oversaturated (white). (D-K) Images are maximum intensity projections.

To get more insights into the cell biology of *D*. *flagrans*, mCherry was C-terminally fused to histone H2B to stain nuclei in living cells. The expression of the fusion protein was under the control of the *D*. *flagrans* *H2B* native promoter. Five of seven transformants showed red fluorescent nuclei, which was confirmed using the nucleic acid dye Hoechst 33342 **(Fig 5A–5C)**. Genomic integration of the construct was verified using PCR. The expression of H2B-mCherry revealed multinucleated conidia and chlamydospores **(Fig 5D–5G)**. Interestingly, trap cells and hyphal compartments contained several nuclei **(Fig 5H–5J)**. In addition, the expression of H2B-mCherry enables to monitor the infection inside the nematode, in which nuclei were visualized by GFP **(Fig 5K)**.

### Analysis of the putative effector protein PefB and establishment of a secretion assay

We used quantitative real time PCR analysis (qRT PCR) to identify genes that are up-regulated during infection of *C. elegans* (24 h co-incubation of the two organisms to induce trap formation) and thus could play a role in virulence. One candidate, gene DFL_009915 (**p**utative **ef**fector protein B, pefB) was chosen for further analyses **(Fig 3B)**. Biological replicates for the expression analysis varied significantly, with 16.1 ± 2.1 (mean ± SD, n = 3), 31.7 (± 4.1) and 81.1 (± 10.4) fold changes, respectively. The relative expression with gamma actin as reference gene showed no expression of *pefB* in the uninduced state **(Fig 3B)**. The large variance between replicates depends on differences in the number of traps and the number of worms trapped at certain times.

The 274 amino acid long protein has a putative N-terminal signal peptide (SP) and a putative nuclear localization signal (NLS) **(Fig 3C)**. The corresponding gene does not contain introns. The protein is rich in serine (19.7%) and arginine (15.3%) and is highly positively charged (isoelectric point = 12.02). PefB does not contain any conserved domains. Analysis of the protein with the “Rapid Automatic Detection and Alignment of Repeats” (RADAR) [48] tool revealed three repeat structures within the protein (**Fig 3C**). A BLAST search resulted in only two hits, revealing 47% identity to the hypothetical A. oligospora gene AOL_s00006g518 (E-value = E-5^−21^) and 48% identity to a 168 amino acid long region of the 481 amino acid long hypothetical Paramormyrops kingsleyae serine/arginine-rich splicing factor 6-like-protein (E-value = 0.22)(XP_023660799.1).

**Fig 6:**
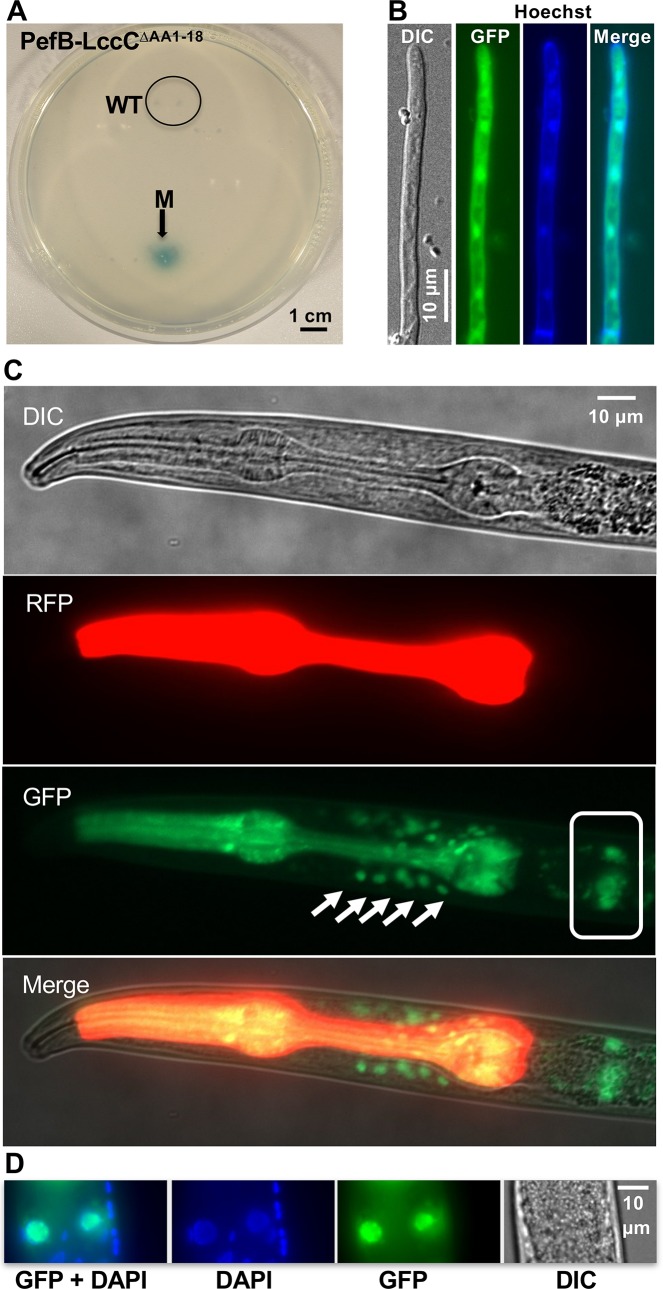
Analysis of the putative effector PefB. **(A)** Secretion of the PefB-laccase fusion protein led to blue-green colored colonies on LNA plates containing 1mM ABTS. The wild-type strain remained uncolored (circle). **(B)** The N-terminal GFP fusion protein of PefB without signal peptide (GFP-PefBΔSP) localized in the cytoplasm and nuclei of *D*. *flagrans*. Nuclei were visualized using Hoechst 33342. **(C)** A C-terminal GFP fusion protein of PefBΔSP expressed in *C*. *elegans* shows nuclear localization. The gene was expressed using the *elt-3* promoter. The cells indicated with the arrows are probably glia and neuronal cells. The picture was overexposed to see the nuclei. The pharynx of *C*. *elegans* shows red fluorescence due to the expression of the marker plasmid. In addition to the weakly stained nuclei on the side of the pharynx, large nuclei of intestinal cells below the pharynx showed the GFP signal (boxed area). **(D)** Another individual of *C*. *elegans* was stained with DAPI. In intestinal nuclei GFP and DAPI signals co-localized.

Since secretion is one main criterium for effector proteins, or protein-based virulence factors, a laccase-assay was established for *D*. *flagrans* to prove the functionality of the SP. PefB was fused to the *A*. *nidulans* laccase C (LccC) lacking its own SP [49]. Laccases catalyze the oxidation of artificial substrates such as ABTS (2,2’-azino-bis-(3-ethylthiazoline-6-sulfonate) in the medium to the more stable state of the cation radical. The enzyme activity therefore correlates with an intense blue-green color in the presence of ABTS. Hygromycin-resistant *D*. *flagrans* transformants indeed showed a positive reaction **(Fig 6A)**. Genomic integration of the construct was verified using PCR. These results show that the described secretion assay is a suitable method for the verification of predicted protein secretion. The laccase may also be used for other purposes in *D*. *flagrans*.

In order to test the functionality of the NLS, the protein (without the signal peptide) was N-terminally fused with GFP and expressed in *D*. *flagrans*. The GFP-PefB fusion protein was visible in the cytoplasm and accumulated in nuclei **(Fig 6B)**. Nuclear localization in *D*. *flagrans* should however, not be the natural residence of PefB, because the protein should be secreted during growth in *C*. *elegans*. Therefore, we tested whether the NLS was functional in C. elegans host cells **(Fig 6C)**. A C-terminal PefB-GFP fusion protein without signal peptide was expressed in *C. elegans*. Similar to the localization of PefB in *D*. *flagrans*, PefB-GFP localized in spots most likely corresponding to nuclei around the terminal bulb of the pharynx. In the case of the two large nuclei below the pharynx we were able to co-localize the protein with DAPI. The other green spots could not be co-localized, probably because the GFP signal was too weak after fixation for DAPI staining. The smaller spots are probably nuclei of neurons or glia cells, whereas the large ones are most likely from intestinal cells (http://wormatlas.org). Future experiments have to reveal if these are the target cells under natural conditions when *D*. *flagrans* colonizes the worm. Nevertheless, the experiment clearly shows that the NLS is functional in *C*. *elegans*.

### Intercellular communication as a prerequisite for trap formation

One fascinating aspect of *D*. *flagrans* and *A*. *oligospora* is the ability to form three-dimensional adhesive networks consisting of hyphal rings to trap nematodes. From the cell biological point of view this is not an easy task, since a network is the result of multiple cell fusions (anastomoses) of hyphal loops [50]. During trap formation a first loop develops as a branch on a vegetative hypha. The loop mostly consists of three hyphal compartments. Prior to ring closure and the fusion of the hyphal tip with the basal hypha, a small hyphal peg is growing towards the trap-forming hypha, indicating intercellular communication. Such a mechanism of cell-to-cell signalling is observed in many filamentous fungi during germling fusion or hyphal anastomosis. A mitogen-activated protein kinase (MAPK) cascade, homologous to the pheromone response pathway of *Saccharomyces cerevisiae*, plays a central role in this communication [51]. In order to investigate the role of hyphal anastomoses during trap formation, similarity searches against various proteins of the N. crassa signalling cascade allowed the reconstruction of the pathway in *D*. *flagrans* **(S7 Fig, S13 Table)** [52].

To test the hypothesis that hyphal anastomosis and cell-to-cell communication is a prerequisite for trap formation, an orthologue of the *N*. *crassa* hyphal anastomosis gene soft (so) was investigated. The open reading frame of the corresponding *D*. *flagrans* gene (DFL007867) consists of 3867 base pairs (bp) with four introns (accession no. MK034754). The introns with lengths of 53 bp, 55 bp, 57 bp, and 60 bp respectively, were confirmed by cDNA cloning and sequencing. They were also in accordance with RNAseq data. The gene was named sofT. We are using the three-letter gene name code in *D*. *flagrans*. The SofT protein consists of 1213 amino acids with a predicted molecular mass of 133 kDa. SofT displayed 56% identity with the SO protein of N. crassa, between 44 and 60% identity with *Aspergillus oryzae*, *Epichloe festucae*, *Dre*. *coniospora*, *P*. *anserina*, *A*. *brassicicola*, *F*. *verticillioides*, *F*. *oxysporum*, and 94% with the putative *A*. *oligospora* orthologue (AOL_s00076g148). All SofT proteins harbour a conserved WW domain, which is characterized by two conserved tryptophan (W) residues spaced by 20 to 22 amino acids.

**Fig 7:**
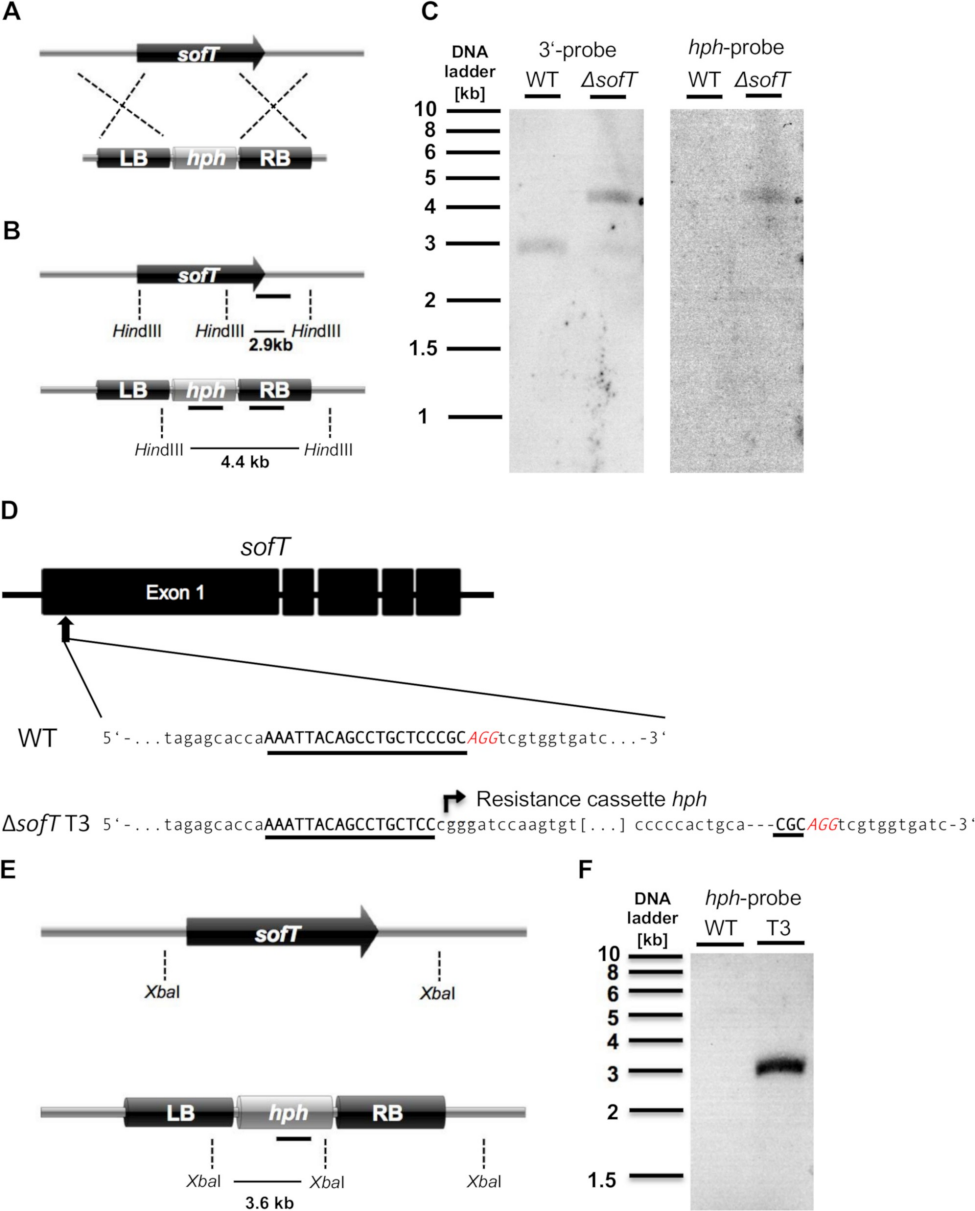
Targeted deletion of the sofT gene in *D*. *flagrans* using homologous recombination or Cas9 RNP. **(A)** Deletion of the *sofT* gene using homologous recombination. (**B)** Scheme of the *sofT*-gene locus from wild type (upper panel) and the *ΔsofT* mutant (lower panel). In the *ΔsofT* strain the ORF (3.8 kb) is replaced by the 1.8 kb hygromycin resistance gene *hph*. Two 1 kb probes and the restriction enzyme *Hin*dIII were used for Southern analyses. **(C)** Confirmation of the *ΔsofT* mutant using Southern blot analysis. Genomic DNA was digested using *Hin*dIII. The 3‘-probe hybridized with a 2.9 kb fragment in wild-type, while in contrast it hybridized with a 4.4 kb fragment in the ΔsofT mutant. The membrane was stripped and hybridized with a *hph*-probe yielding in a single 4.4 kb fragment only in the *ΔsofT* mutant. **(D)** Deletion of the *sofT* gene using the Cas9 RNP transformation system. The 20bp target sequence in the *sofT* gene is underlined and located in the first exon of the gene. The PAM sequence (AGG) is shown in red. The *ΔsofT* mutant T3 showed insertion of the hygromycin-B resistance cassette three nucleotides upstream of the PAM sequence. **(E, F)** Single integration of the hygromycin-B resistance cassette in the *ΔsofT* mutant T3 was verified using Southern analysis. Genomic DNA of wild-type and the *ΔsofT* mutant T3 was digested using the *Xba*I restriction enzyme resulting in a 3.6 kb fragment after hybridization with the *hph*-probe.

To investigate the biological function of SofT during trap formation, we established a gene knock-out system for *D*. *flagrans* based on homologous recombination **(Fig 7A and 7B)**. 20 transformants were obtained growing on PDA with 100 μg/ml hygromycin-B. They were screened via PCR and Southern blot analysis **(Fig 7C)**. One transformant showed the expected integration.

In order to confirm the phenotype of the ΔsofT-deletion strain, and to establish the CRISPR/Cas9 system in *D*. *flagrans*, we aimed at achieving gene inactivation with this system. First, we tried the plasmid-based approach established for *A*. *nidulans*, *A*. *alternata* and other fungi [53, 54]. However, no stable transformants were obtained. Next, we tested simultaneous transformation of Cas9 ribonucleoproteins (RNPs) and the linear resistance gene hph based on recent reports [55–57]. The sgRNA cleavage site was located in the first exon of the sofT gene (**Fig 7D**). To avoid off-target cleavage, the uniqueness of the protospacer region and the protospacer adjacent motif (PAM) sequence were analysed in the *D*. *flagrans* genome using BLASTN. In total four transformants were obtained and the region surrounding the protospacer region was sequenced to analyse the mutations. In three of the four transformants, the linear resistance gene hph was inserted into the cleavage site. In addition, small deletions of one or two nucleotides were detected in the ΔsofT mutants. Interestingly the 5’ and 3’ ends of the inserted hph gene were slightly modified by nucleotide deletions. Transformant T3 showed single integration of the hph gene into the genome as shown by Southern blot analysis and was used for further experiments **(Fig 7E and 7F)**.

**Fig 8:**
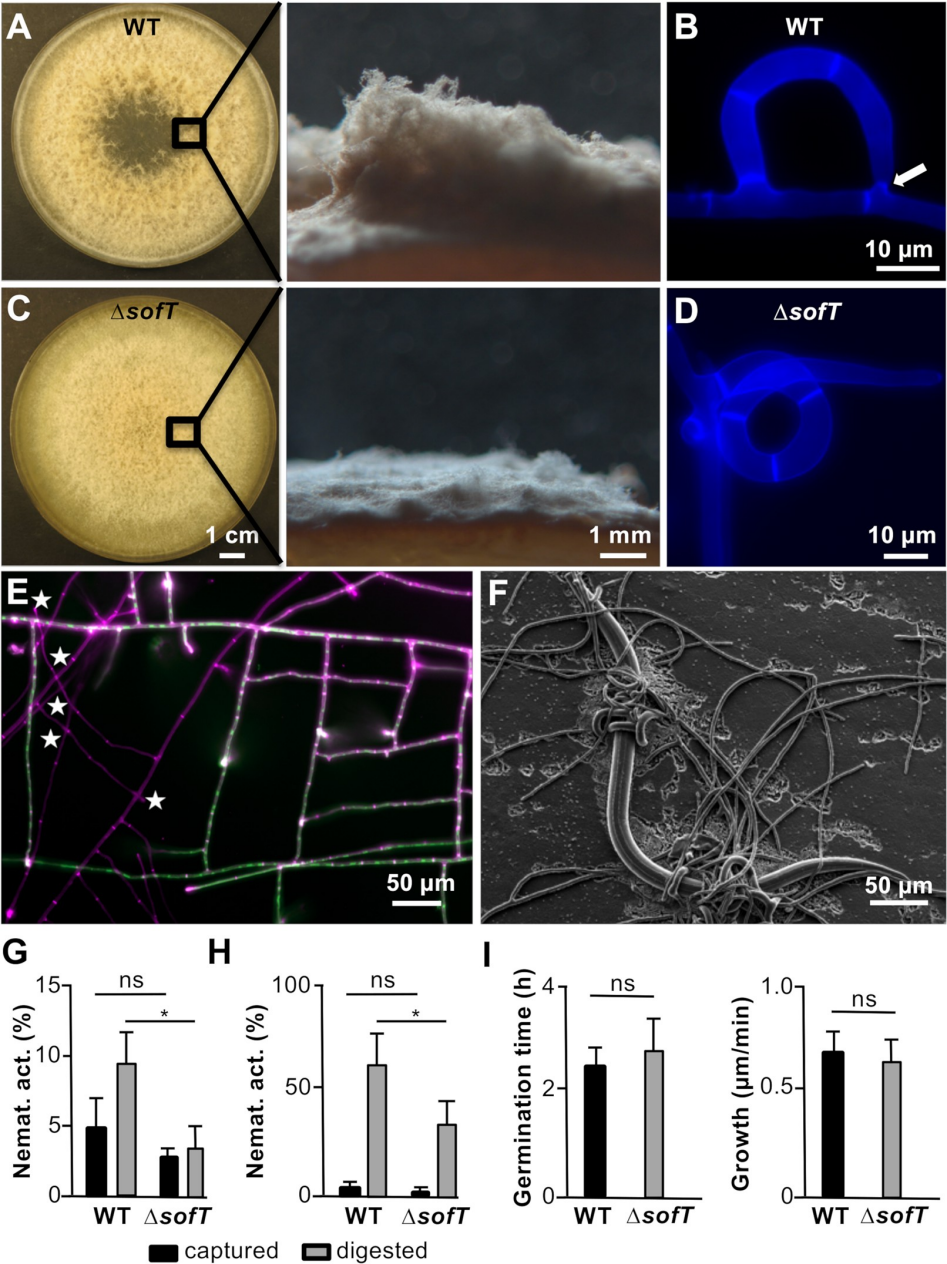
The *ΔsofT* mutation led to the reduction of aerial mycelium, incomplete trap closure, absence of hyphal anastomoses and decrease in virulence. **(A)** Growth of *D*. *flagrans* wild-type on PDA after 7 days. Note the production of aerial mycelium shown by the colony view from the side. **(B)** Prior to ring closure a small hyphal peg (arrow) is growing towards the trap-forming hypha. **(C)** Growth of the *D*. *flagrans* *ΔsofT* mutant on PDA after 7 days. Aerial mycelium was reduced as seen in view of the colony from the side. **(D)** Deletion of the *sofT* gene prevents ring closure during trap formation. **(B+D)** Fungal cell walls were stained using CFW. Images are maximum intensity projections. **(E)** Lack of hyphal anastomoses in the *ΔsofT*-mutant strain T3. Fungal cell walls were stained using CFW (magenta). The mutant strain (stained only in magenta) was grown on the same slide as the control strain (wild-type background) expressing cytoplasmic GFP (stained in green + magenta) for 24 h. Hyphal anastomoses were absent despite close physical contact of the hyphae in the *ΔsofT* mutant (stars) compared to numerous hyphal anastomoses in the control strain. **(F)** Complex formation of trapping networks and trapping of *C*. *elegans* by the *ΔsofT* mutant. **(G, H)** Comparison of the nematicidal activity (in %) of *D*. *flagrans* wild type and the *ΔsofT* mutant on LNA after 24 and 36 h, respectively. The nematicidal activity is divided into captured (alive, black bar) and digested (dead, grey bar) state of the nematode. Significance was tested using an unpaired t test (P < 0.05: ns = not significant; * = significant). **(I)** Comparison of the germination time and fungal growth of *D*. *flagrans* wild-type and the *ΔsofT* mutant. Significance was tested using an unpaired t test (P < 0.05).

All ΔsofT mutant strains grew with less aerial hyphae and trap formation was disturbed at the step of ring closure, resulting in spiral hyphae **(Fig 8A–8D).** Anastomoses in normal vegetative hyphae were completely inhibited **(Fig 8E)**. Despite the observed defects, the *ΔsofT*-deletion strain formed complex three-dimensional trapping networks and was still able to trap C. elegans **(Fig 8F)**. Next, the mutant generated by the CRISPR/Cas9 system was transformed with a plasmid containing the full-length *sofT* gene including the regulatory regions (promoter and terminator). In two out of six transformants wild-type phenotype was restored. The integration of the construct was verified by PCR. A virulence assay was performed to further investigate the lack of SofT on the virulence of *D*. *flagrans*. The nematicidal activity of the fungi was divided into captured (alive) or already digested (dead) nematodes after 24 and 36 h **(Fig 8G and 8H)**. After 24 h the wild-type captured 10 ± 4 (mean ± SD) % while 19 ± 5% were already digested. In the ΔsofT mutant 5 ± 2% (P = 0.1658 unpaired t test) were captured while 7 ± 4% (P = 0.0235 unpaired t test) were already digested after 24 h. After 36 h in the wild-type 5 ± 2% were captured while 65 ± 14% were already digested. In the ΔsofT mutant 1 ±1% (P = 0.0514 unpaired t-test) were captured while 35 ± 12% (P = 0.0476 unpaired t-test) were already digested. To exclude that the attenuated virulence was due to vegetative growth or fitness defects, germination time and fungal growth were analysed of the mutant **(Fig 8I)**. Wild-type conidia germinated on LNA after 2.5 ± 0.4 h (mean ± SD, n = 6) while ΔsofT conidia germinated after 2.9 ± 0.6 h (P = 0.1658 unpaired t test, n = 6). Growth of wild-type hyphae on LNA was 0.71 ± 0.09 μm/s (n = 6) while the growth of the *ΔsofT* hyphae was 0.68 ± 0.08 μm/s (P = 0.5209 unpaired t test, n = 6). Hence, germination and hyphal growth were not very different in wild type and mutant. The reduced virulence of the *ΔsofT* mutant may be due to decreased stress tolerance. It was shown that the A. oryzae SO orthologue AoSO forms aggregates at the septal pore in response to physical stress, low and high temperature and extreme acidic or alkaline pH [58]. In addition, the AoSO mutant strain has defects in stress granule formation during exposure to heat stress [59]. Thus, the *ΔsofT* *D*. *flagrans* mutant may be more sensitive towards stress. The innate immune response of *C*. *elegans* is accompanied by an increase of reactive oxygen species (ROS), to cope with bacterial and fungal pathogens attacking the worm from the intestine or the cuticle [60]. In the insect pathogenic fungus Metarhizium acridum, it has recently been shown that a catalase peroxidase, MakatG1, is expressed specifically on the host cuticle [61]. *D*. *flagrans* SofT could play a role in the signalling pathway of the ROS-induced stress response.

## Materials and methods

### Cultivation of the organisms

*D*. *flagrans* (CBS 349.94) was obtained from the CBS-KNAW culture collection (The Netherlands) and was cultured at 28°C on PDA. Cultivation and synchronization of *C. elegans* was performed according to the worm book (doi/10.1895/wormbook.1.101.1). X. index was isolated from fig tree roots using a modified Baermann funnel [29].

### Trap induction

To induce trap formation around 1x10^4^ *D*. *flagrans* spores were inoculated on thin LNA (pH 6.5) and around 100 individuals of C. elegans were added. Co-incubation was carried out at room temperature in darkness for at least 6 hours. Experiments with *X. index* were performed on LNA plates where 10 individuals were co-incubated with 5x10^4^ *D*. *flagrans* spores at room temperature.

To induce trap formation for RNA extraction 10^6^ *D*. *flagrans* spores were incubated on LNA with 1% glucose covered with a cellophane membrane for 24 h at 28°C. 10^4^ individuals of a mixed *C. elegans* population were added to the membrane and co-incubated for 24 h to induce trap formation. The uninduced group was treated equally without the addition of nematodes.

### Glycogen and nucleic acid staining

Glycogen of *D*. *flagrans* hyphae, conidia and chlamydospores was stained with Lugols’s iodine (Roth). Nuclei were stained using Hoechst 33342 (5 μg/ml working concentration, incubation time 5 min, Invitrogen, Karlsruhe, Germany) or Vectashield antifade mounting medium with DAPI (Vector Laboratories, Peterborough, UK). C. elegans nuclei were stained using Vectashield antifade mounting medium with DAPI after fixation with 95% ethanol [62].

### Cell wall staining

The cell wall was stained with Calcofluor white M2R (fluorescent brightener 28, Sigma-Aldrich). A 1% (wt/vol) stock solution was prepared by dissolving CFW in 0.5% (wt/vol) KOH and 83% (vol/vol) glycerol. The stock solution was diluted 1:1000 (dH_2_O) and incubated for 5 min to stain the cell wall.

### Genome sequencing, assembly and analysis

For extraction of genomic DNA, *D*. *flagrans* was grown on CM-agar for 7 days. Spores were collected in 100 ml CM liquid medium and incubated at 28°C for 24 h at 180 rpm. Protoplasts were generated as described above and genomic DNA was extracted using Genomic DNA Buffer Set (Qiagen) and Genomic-tip 20/G (Qiagen) according to the manufacturer’s instruction. In total 12 μg of DNA were sent for sequencing.

The *D*. *flagrans* genome was sequenced using the PacBio RS technology (Pacific Biosciences, Menlo Park, CA, USA) with libraries prepared with the SMRTbell template prep kit 1.0 (Pacific Biosciences). The sequencing runs and assembly of the libraries were carried out by GATC biotech (Konstanz, Germany). The Whole Genome Shotgun project has been deposited at DDBJ/ENA/GenBank under the accession SAEB00000000. The version described in this paper is version SAEB01000000. Raw reads were deposited at SRA under the accession SRR8400569.

### RNA-seq

Total RNA was extracted using the E.Z.N.A. Fungal RNA Kit (OMEGA bio-tek) according to the manufacturer’s protocol. Trap induction for the zootrophic stage was induced as described above using LNA without glucose. For saprotrophic growth, around 5x10^6^ *D*. *flagrans* spores were inoculated in PDM in 3.5 cm petri dishes and were cultivated for 48 h at 28°C. The extracted RNA was checked using a NanoDrop 2000 (Thermo, CA, USA), and the RNA concentration and integrity were assessed using the RNA Nano 6000 Assay Kit of the Agilent Bioanalyzer 2100 system (Agilent, CA, USA) to ensure that the RNA Integrity Number (RIN) values were above 7.0. Oligo (dT) beads were used to isolate poly(A) + mRNA, which was fragmented to 250 bp. Fragmentation of the RNA and reverse transcription of double-strand cDNA (ds cDNA) was done by using N6 random primers. The synthesized cDNA was subjected to end-repair and then 3′ adenylated. Adaptors were ligated to the cDNA ends. After amplification each cDNA library was sequenced in a single lane of the BGISEQ-500 system using paired-end sequencing with lengths of 100 bp according to the manufacturer’s instructions at the Beijing Genomics Institute (Beijing, China). 23,941,723 reads were generated and 91.79% mapped to the *D*. *flagrans* genome and 88.84% had a unique match. 85.28% of the reads were mapped to genes, 85.01% had a unique match.

### Gene prediction, annotation and protein classification

Gene models were predicted with the ab initio predictor BRAKER2 [63–65]. BRAKER2 was implemented with GeneMark-EX and subsequently with AUGUSTUS. Additionally, RNAseq was included to improve intron predictions.

The *D*. *flagrans* proteome was blasted against the Swissprot using an E-value limitation of E-10^−5^. *A. oligospora* was used as database for BLAST searches of *D*. *flagrans* using an E-value limitation of E-10^−5^. Protein subcellular localization was predicted using WoLF Psort [66].

### Orthologous proteins and orthologous protein families

The proteomes of *A*. *oligospora*, *Dre*. *coniospora*, *Da*. *haptotyla*, and *Dr*. *stenobrocha* were downloaded from NCBI and blasted against the *D*. *flagrans* proteome using at least 70% amino acids identity and 80% coverage to *D*. *flagrans* proteins in order to identify orthologous proteins. On the other hand, orthologous protein families were determined among *D*. *flagrans*, *A*. *oligospora*, *Da*. *haptotyla* and *Dr*. *stenobrocha* in OrthoVenn. Orthologous gene pairs were considered on the basis of amino acid sequence similarities sharing up to 50% of the total length of the shorter gene being analysed (BLASTP, threshold E-value ≤ 1E-10^−5^).

### Secretome generation

Signal peptides were predicted using SignalP v4.1 [67]. Secreted proteins were further predicted using WoLF PSORT, a protein localization prediction program trained with fungal data. Proteins that may be secreted but probably membrane bound were filtered out using TMHMM v2.0 [68, 69]. Proteins combining these criterions were joined to generate the secretome. The tools were used at Galaxy Uni-Freiburg [70, 71] and filtration was implemented by a custom Perl script. Nuclear localization was predictied using NLStradamus [72]. The secretomes of *A. oligospora* (ADOT00000000.1), *Fusarium graminearium* (AACM00000000.2), *Aspergillus nidulans* (AACD00000000.1), *D. coniospora* (LAYC00000000.1), *Dr. stenobrocha* (ASQI00000000.1), *Dr. stenobrocha* (AACP00000000.2), *Neurospora crassa* (AABX00000000.3), *Dactylellina haptotyla* (AQGS00000000.1), *Penicillium brasilianum* (CDHK00000000.1), *Aspergillus fumigatus* (AAHF00000000.1), *Candida albicans* (GCA_000182965.3), *Candida galabrata* (GCF_000002545.3), *Alternaria alternata* (LXPP00000000.1), *Magnaporthe oryzae* (AACU00000000.3), *Trichoderma reesei* (AAIL00000000.2), *Pochonia chlamydosporia* (LSBJ00000000.2), *Botrytis cinera* (GCA_000143535.4), *Metarhizium acridum* (ADNI00000000.1), *Metarhizium album* (Metarhizium album), *Hirsutella minnesotensis* (JPUM00000000.1), *Cordyceps militaris* (AEVU00000000.1) proteomes were generated on the same way as *D*. *flagrans*. Venn diagrams were generated using VennDiagram package in R.

### Protoplast transformation of *D*. *flagrans*

The transformation system was adapted from [45]. Briefly 1x10^8^ spores were inoculated in 150 ml PDM and incubated for 24 h at 28°C and 180 rpm. The mycelium was harvested and washed with MN solution (0.3 mol/l MgSO_4_, 0.3 mol/l CaCl_2_) and ca. 0.5 g of mycelia (wet weight) was collected and suspended in 5 ml MN buffer containing 4 mg/ml kitalase (Fujifilm Wako Chemicals) and 20 mg/ml VinoTaste Pro (Novozymes) followed by incubation at 30°C, 70 rpm for 3 h. Quality of protoplasts was checked using light microscopy. Subsequently undigested mycelium was removed by filtering the protoplast through 3-layer of miracloth tissue. The protoplasts were precipitated at 3350 g for 15 min. After carefully removing the supernatant the protoplasts were washed with 50 ml KTC buffer (1.2 mol/l KCl, 10 mmol/l Tris-HCl pH 7.5) and the resulting pellet was resuspended in 500 μl KTC.

For transformation 100 μl protoplasts (5x10^6^) were mixed with 5–8 μg of DNA and incubated for 2 min on ice. Then 1 ml of PTC (10 mmol/l Tris-HCl pH 7.5, 50 mmol/l CaCl_2_, 60% W/V PEG6000) was added and incubated at room temperature for 20 min. 10 ml PDSSA (24 g/l potato dextrose broth, 0.6 mol/l sucrose, 0.3 g/l peptone, 0.3 g/l tryptone, 0.3 g/l yeast extract, 14 g/l agar) (lukewarm) was added to the transformation mix and was poured onto PDA plates supplemented with 100 μg/ml hygromycin-B, 60 μg/ml nourseothricin, or 120 μg/ml geneticin (G418), respectively and incubated at 28°C for 4–7 days.

### sgRNA design and RNP formation

The sgRNA for the sofT gene mutation was synthesized using the EnGen sgRNA Synthesis Kit (New England Biolabs, Frankfurt). Ribonucleoprotein (RNP) formation was carried out at 37°C for 20 min with 4 μl unpurified sgRNA, 10 μl Cas9 (200nM), 5 μl 10x Cas9 Nuclease Reaction Buffer and DEPC-treated water in 50 μl total volume. For protoplast transformation, the linear resistance gene hph was amplified by PCR, subsequently purified using FastGene Gel/PCR Extraction kit (Nippon Genetics) and lastly about 2 μg purified PCR-product were added to the transformation-mix. The transformation was carried out as described.

### Plasmid construction

To create a C-terminal mCherry fusion of the histone H2B under its natural promoter, a 1.6 kb fragment including the ORF of DFL_000203 (0.6 kb without stop codon) + 1 kb of the 5’ region and a 1 kb fragment 3’ downstream of the locus were amplified by PCR, using *D*. *flagrans* genomic DNA as template. The mCherry gene from plasmid 12A_pNDH-OCT [59] and the hph from plasmid pFC332 [53] were amplified by PCR. All fragments were assembled into the pJET1.2 vector (Thermo Fisher, digested with EcoRV) using the NEBuilder HiFi DNA Assembly Cloning Kit (New England Biolabs, Frankfurt) resulting in plasmid pVW23.

For the N-terminal GFP fusion protein of DFL_009915 (PefB) without the signal peptide the gene was amplified with a forward primer starting at bp 60 and containing an AscI restriction site and start codon and a reverse primer containing a PacI restriction site. The amplicon was cut with the respective enzymes and ligated into a plasmid that contains the hygromycin-B resistance cassette hph [53] and the GFP gene under control of the constitutive A. nidulans oliC promotor and the glucanase terminator of B. cinerea (oliC(p)::GFP::pefB::gluC(T)) with a pJET1.2 backbone resulting in the plasmid pIH02.

For the ABTS-assay, the modified vector pOF018 was used for the expression of the A. nidulans lccC gene (AspGD identification AN5397) under the constitutive A. nidulans glyceraldehyde-3-phosphate dehydrogenase (gpdA) promoter and as positive control for laccase activity [71]. For the selection of *D*. *flagrans* the hygromycin B resistance cassette was amplified using pFC332 [53] as template and inserted into the vector after linearization with XbaI, using the NEBuilder HiFi DNA Assembly Cloning Kit [73]. The gene sequence of pefB was amplified with primers adding overlapping regions to the vector. The vector backbone was amplified via PCR with primers binding after the signal peptide sequence (60 bp) to remove the laccase signal peptide. The pefB gene sequence was fused to the 3’ end of the laccase C using the NEBuilder HiFi DNA Assembly Cloning Kit.

For the expression of PefB in *C*. *elegans*, the open reading frame was cloned, without the predicted signal peptide, into the pLZ29 backbone [74] using the Gibson assembly method [73]. The resulting assembly made an in-frame C-terminally tagged GFP fusion protein under expression of the ubiquitous eft-3 promoter.

The *sofT* gene was deleted by homologous recombination. Two kb flanks homologous to the 5’ and 3’ regions of the sofT gene were amplified by PCR, using *D*. *flagrans* genomic DNA as template. The hygromycin-B resistance cassette hph was amplified using pFC332 [53] as template. The PCR-fragments were assembled into the pJET1.2 vector (Thermo Fisher, digested with EcoRV) using the NEBuilder HiFi DNA Assembly Cloning Kit (New England Biolabs, Frankfurt). The fragments were amplified with Phusion polymerase (NEB) using the manufacturers recommended protocol and contained 25 bp overlapping regions to the neighbouring fragment. Standard protocols were used for E. coli transformation and plasmid isolation [75].

To confirm the *sofT* introns, ca. 5x10^6^ *D*. *flagrans* spores were inoculated in PDM in 3.5 cm petri dishes and were cultivated for 48 h at 28°C. The mycelium was harvested and frozen immediately in liquid nitrogen. RNA isolation was performed using E.Z.N.A. Fungal RNA Kit (OMEGA bio-tek) according to the manufacturer protocol. To remove DNA the RNA was treated with TURBO DNA-free kit (Invitrogen, Karlsruhe, Germany). cDNA synthesis was performed using the SuperScript Double-Stranded cDNA Synthesis Kit (Thermo Fisher Scientific, Waltham, MA, USA) according to the manufacturer protocol. The sofT coding sequence was amplified by PCR using *D*. *flagrans* WT cDNA as template and assembled into the pJET1.2 vector. Sequencing was carried out by MWG-Eurofins.

To reintroduce the functional *D*. *flagrans* *sofT* gene into the ΔsofT deletion strain, the whole sofT gene, including its 1-kb upstream and 0.5 downstream regulatory regions, was amplified via PCR, using *D*. *flagrans* genomic DNA as template. To select for transformants the geneticin G418 resistance cassette was used. In a cloning step, the G418 resistance cassette was assembled between a new trpC(p) promoter and a new trpC(t) terminator fragment of pVW23 using the NEBuilder HiFi DNA Assembly Cloning Kit. Lastly, the new G418 resistance cassette and the *sofT* complement were assembled into the pJET1.2 vector (Thermo Fisher, digested with EcoRV) using the NEBuilder HiFi DNA Assembly Cloning Kit.

All primers used for plasmid constructions and sequencing are listed in **S14 Table**.

### Generation of transgenic *C*. *elegans* strains

The plasmid harboring the pefB::GFP fusion construct was injected at a concentration of 50ng/ul into wildtype worms (N2) with a pharyngeal co-injection marker (myo-2p::tdTomato) and co-injection marker positive transformants were selected.

### RNA extraction and quantitative RT-PCR

For RNA extraction the mycelium was scratched off the cellophane membrane and ground in liquid N_2_. Total RNA was extracted with Trizol reagent (Invitrogen, Karlsruhe, Germany). DNase digestion was performed using the Turbo DNA-free Kit (Invitrogen, Karlsruhe, Germany) and the RNA was diluted to 50 ng/μl. The SensiFast SYBR and fluorescein One Step Kit (Bioline, Luckenwalde, Germany) was used for the qRT-PCR analysis on an CFX Connect Real-Time PCR Detection System (Bio-Rad, Munich, Germany). Each reaction mixture contained 0.2 μM primers **(S14 Table)** and 80 ng of RNA in a 25-μl total volume. Melting curve analysis was performed to assess the specific amplification of DNA. Fold changes were calculated using the formula 2^−(ΔΔCt)^, with ΔΔCt being ΔCt (treatment)-ΔCt (control), ΔCt is Ct (target gene)—Ct (actin), and Ct is the threshold cycle. The gamma actin orthologue DFL_002353 was used as internal reference gene for normalization. The qRT PCR was performed using 3 biological replicates.

### ABTS-assay

For the ABTS-assay 1x10^4^ spores of *D*. *flagrans* transformands were grown on a modified low-nutrient-agar (LNA) modified after [73] (KCL 1g/L, MgSO_4_ - 7H_2_O 0.2g, MnSO_4_ - 4H_2_O 0.4mg, ZnSO_4_ - 7H_2_O 0.88mg, FeCl_3_ - 6H_2_O, 3mg, Agar 10g, pH 5.5) containing 1 mM ABTS (2,2‘-azino-bis-[3-ethylthiazoline-6-sulfonate]) at 28°C. Laccase activity was correlated with the formation of blue-green ABTS• visible after 24–48 h.

### Microscopy

For microscopy around 4x10^4^ spores were inoculated on thin LNA and incubated for at least 14 h at 28°C. Conventional fluorescence images were captured at room temperature using a Zeiss Plan-Apochromat 63x/1.4 Oil DIC, EC Plan-Neofluar 40x/0.75, EC Plan-Neofluar 20x/0.50, or EC Plan-Neofluar 10x/0.30 objective attached to a Zeiss AxioImager Z.1 and AxioCamMR. Color images were acquired using the AxioCam 105 color. Images were collected using ZEN 2012 Blue Edition.

Confocal images were captured at room temperature using a Leica HCX PL APO 63x1.4 oil objective attached to a Leica TC SP5 and conventional photomultiplier tube detectors (Leica, Wetzlar, Germany). Kryo-SEM was performed as previously described [72]. Stereomicroscopy was performed using a Zeiss Lumar.V12 with AxioCam HRc and NeoLumar S 1.5x objective. Images were collected using the AxioVision software.

To quantify germination time and hyphal growth of *D*. *flagrans* wild-type and the ΔsofT strain an AxioObserver Z1 inverted microscope employing a 10x/0.30 N.A. objective (Zeiss) was used.

### Virulence assay

To compare the virulence of the *D*. *flagrans* wild-type and the ΔsofT mutant strain 1x10^5^ spores were plated on a petridish (3.5 cm diameter) containing LNA. As bait 200 synchronized C. elegans L2 larvae (strain BAN126) were used and added to the plate and incubated at room temperature. After 24 and 36 h the number of captured or already digested nematodes was counted using a light microscope (Zeiss AxioImager Z.1). The assay was performed with three biological replicates.

### Germination and hyphal growth experiments

To determine the germination time and hyphal growth of the *D*. *flagrans* wild-type and the ΔsofT mutant 1x10^4^ spores were plated on thin LNA. The experiments were carried out at 28°C. The hyphal growth was calculated by kymograph analysis using the Fiji software (https://fiji.sc).

## Supporting information

S1 Text Additional genome features. (DOCX)

S1 Fig Genome structure of *D*. *flagrans*. Circular map displaying genomic features of the *D*. *flagrans* genome. Distinct contigs are depicted using Circos with colored sectors on the outer layer resulting in 36.6 Mb. From outside to inside: **(a)** CDS position, **(b)** Intron position, **(c)** percentage of G+C, **(d)** percentage of GC skew, **(e)** effector genes, **(f)** secretome genes, **(g)** SSP genes. Inner part: non-coding RNA genes, afu (small nucleolar RNAs known in *Aspergillus fumigatus*), rRNAs (black) and small nuclear RNAs (snRNAs, green). The original contig numbers provided by Pacific Biosciences were kept in order to match them with the published sequence.
(PPTX)

S2 Fig Graphical representation of the GO term association data for 6,878 proteins and pathways identified in *D*. *flagrans* (https://chirimoyo.ac.uma.es/bitlab/portfolio/sma3s/). The slices represent the percentage of unigenes identified in the particular category. **(A)** Biological process. **(B)** Molecular function. **(C)** Uniprot pathways.
(PPTX)

S3 Fig Genome-based phylogenomic tree of NTF and other fungi. Bootstrap values are indicated beside the nodes. Orange color indicates the clade grouping of nematode trapping fungi. The proteomes of *Arthrobotrys oligospora* (ADOT00000000.1), *Drechmeria coniospora* (LAYC00000000.1), *Dactylellina haptotyla* (AQGS00000000.1), *Fusarium graminearum* (AACM00000000.2), *Hirsutella minnesotensis* (JPUM00000000.1), *Metarhizium acridum* (ADNI00000000.1), *Metarhizium anisopliae* (AZNF00000000.1), *Magnaporthe oryzae* (AZNF00000000.1), *Pochonia chlamydosporia* (LSBJ00000000.2), *Tolypocladium ophioglossoides* (LFRF00000000.1) and *Trichoderma reesei* (AAIL00000000.2) were downloaded from NCBI and compared using BLASTP against the *D*. *flagrans* proteome. The matches with at least E value < = 1E-100 and at least 70% sequence identity over 85% of the protein lengths were taken as homologous sequences. The set of 93 high-confidence orthologous proteins were concatenated and aligned using Clustal Omega [76] and phylogenetic distances were calculated using the maximum likelihood-based method implemented within MEGA using default parameters. Tree Of Life (iTOL) v3 was used to visualize MEGA output [77].
(PPTX)

S4 Fig Comparison of the proteomes and secretomes of several fungi. 21 fungal secretomes and effectors were predicted and were compared to those of *D*. *flagrans*. All secretomes were generated on the same way as for *D*. *flagrans* (See Materials and methods). The proteomes of *A*. *oligospora* (ADOT00000000.1), *Fusarium graminearium* (AACM00000000.2), *Aspergillus nidulans* (AACD00000000.1), *Dre*. *coniospora* (LAYC00000000.1), *D*. *stenobrocha* (ASQI00000000.1), *Ustilago maydis* (AACP00000000.2), *Neurospora crassa* (AABX00000000.3), *Dactylellina haptotyla* (AQGS00000000.1), *Penicillium brasilianum* (CDHK00000000.1), *Aspergillus fumigatus* (AAHF00000000.1), *Candida albicans* (GCA_000182965.3), *Candida galabrata* (GCF_000002545.3), *Alternaria alternata* (LXPP00000000.1), *Magnaporthe oryzae* (AACU00000000.3), *Trichoderma reesei* (AAIL00000000.2), *Pochonia chlamydosporia* (LSBJ00000000.2), *Botrytis cinera* (GCA_000143535.4), *Metarhizium acridum* (ADNI00000000.1), *Metarhizium album* (AZHE00000000.1), *Hirsutella minnesotensis* (JPUM00000000.1), *Cordyceps militaris* (AEVU00000000.1) were downloaded from NCBI. Venn diagrams were generated using the VennDiagram package in R. **(A)** The secretomes and putative effectors of 22 fungi are compared. The purple color presents secretome proteins frequency and grey color presents effectors protein frequency. **(B)** Comparison of nematophagous, insect pathogens, human pathogens, plant pathogens and saprophytic fungi proteome sizes relative to the secretome sizes. Lines in grey, red and blue represent limits of 1%, 5% or 8% of secreted proteins respectively.
(PPTX)

S5 Fig Clustering of orthologous proteins exclusive to *D*. *flagrans* common in five chlamydospore-forming fungi. The OrthoVenn diagram shows the clustering of orthologous proteins exclusive to *D*. *flagrans* (not found in *A*. *oligospora*) in the five chlamydospore forming fungi *Botrytis cinerea*, *Cryptococcus neoformans*, *Fusarium oxysporum* and *Pochonia chlamydosporia*. The comparison of the proteomes of *D*. *flagrans* and *A*. *oligospora* resulted in 591 proteins exclusive to *D*. *flagrans* that could represent candidates involved in chlamydospore 46 formation. These proteins were compared to the proteomes of five chlamydospore forming fungi and then clustered using OrthoVenn. The 591 *D*. *flagrans* proteins formed 81 clusters including 8 clusters that can be found in five chlamydospore forming fungi.
(PPTX)

S6 Fig Comparison of nematode-trapping fungi secretome proteins. **(A)** Venn diagram of *D*. *flagrans*, *A*. *oligospora* and *D*. *haptotyla* secretomes, 157 orthologous proteins are shared. **(B)** Venn diagram of orthologous gene clusters of *D*. *flagrans*, *A*. *oligospora*, *Da*. *haptotyla* and *D*. *stenobrocha*. The four nematode trapping fungi share 139 clusters.
(PPTX)

S7 Fig Germling and hyphal fusion pathway in *D*. *flagrans*. The pathway was reconstructed using the *N*. *crassa* model [52]. A so far unknown signaling molecule is released by the signal-emitting cell, probably by exocytosis, in a SO-dependent manner. The signal is perceived by a so far unknown receptor of the signal-receiving cell. The binding leads to the assembly and activation of the MAK-2 module at the plasma membrane, which causes phosphorylation of MOB-3 of the 47 STRIPAK complex, and subsequent entry of MAK-1 into the nucleus. Inside the nucleus gene expression of cell fusion-relevant genes is controlled by the MAK-2 activated transcription factor PP-1. The activation of MAK-2 involves the production of reactive oxygen species by the NADPH oxidase (NOX) complex. MAK-2 is assembled at the plasma membrane with the scaffold protein HAM-5, the adaptor protein STE50 and its two upstream kinases MEK-2 and NRC-1 in an oscillating manner. The *D*. *flagrans* orthologues are displayed as numbers without the DFL_ prefix.
(PPTX)

S1 Table Length and mean coverage of *D*. *flagrans* contigs. The length of the contigs is given in bp. The mean coverage is calculated for each contig and the last row is the sum of length and mean coverage of all contigs.
(XLS)

S2 Table ATGC repartition in each contig. The length of the contigs is given in bp. The rest of the columns show the percentage of nucleotide usage in each contig.
(XLS)

S3 Table Genome size, number of predicted protein-coding genes, G + C content and best Blast top hit homology between *D*. *flagrans* and other 8 fungal genomes. The columns show: Genome size in Mb, percentage of GC content, number of proteins, best blast top hit (orthologous proteins), the life style and NCBI project accession number.
(XLS)

S4 Table BlastP result of *D*. *flagrans* proteins analyzed using the Swissprot database. Columns show: qseqid: query accession dot version, sseqid: subject accession dot version (database hit), pident: percentage of identical matches, length: alignment length, mismatch: number of mismatches, gapopen: number of gap openings, qstart: start of alignment in query, qend: end of alignment in query, sstart: start of alignment in subject (database hit), send: end of alignment in subject (database hit), evalue: expectation value (E-value 10 E-5), bitscore: bit score.
(XLS)

S5 Table BlastP result of *D*. *flagrans* proteins compared with *A*. *oligospora* proteins with e-values below 10 E-5. The columns are the same as in S4 Table.
(XLS)

S6 Table *D*. *flagrans* mitochondrial genes and their annotation. Mitochrondrial proteins and their corresponding description in the InterProScan database.
(XLS)

S7 Table Annotated *D*. *flagrans* proteome using Pfam. All *D*. *flagrans* proteins with Pfam or Interpro domains are depicted. Columns: proteins assigned with locus-tag, Pfam domain, description from Pfam database, E-value, Interpro domain, description from Interpo database.
(XLS)

S8 Table Comparison of the Pfam domains in 4 NTF. Pfam domains and the corresponding numbers found in *D*. *flagrans*, *A*. *oligospora*, *Da*. *haptotyla* and *Dre*. *coniospora*.
(XLS)

S9 Table PHI base and the corresponding number of genes in *D*. *flagrans*. *D*. *flagrans* proteins blasted against the Pathogen–Host Interaction database (PHI). Columns: PHI accession number, number of proteins found in *D*. *flagrans* and virulence effect level.
(XLS)

S10 Table Pathogenesis-related genes in several fungal genomes. Comparison between proteins (transporter, signaling, oxidation, transcription regulation, metabolism and some others) from the PHI database between *D*. *flagrans*, *A*. *oligospora*, *Da*. *haptotyla*, *Dre*. *coniospora* and *Pochonia chlamydospora*. Columns: PHI accession number, description of the protein with virulence effect level, GO ontology annotation, the number of the corresponding protein in the corresponding fungus.
(XLS)

S11 Table Detailed annotations of the *D*. *flagrans* secretome based on the Sma3s tool and InterProScan. Columns: protein accession, protein name (when found in Uniprot 90), description of the protein, enzyme reference, GO ontology, keyword, IPR domain and IPR description.
(XLS)

S12 Table Secretome and effector comparison between several fungi. 21 fungi were compared at the proteome, secretome and effectors level. Columns: name of the fungus, number of proteins, number of proteins in secretome, % of secretome compared to proteome, number of protein effectors, % of effectors in secretome.
(XLS)

S13 Table Genes involved in germling/hyphal fusion in *N*. *crassa*, *Sordaria macrospora* and orthologues in *D*. *flagrans*. The orthologues and accession numbers of *N*. *crassa* and *S*. *macrospora* are shown. The *D*. *flagrans* orthologues were identified by BLAST search against *N*. *crassa*.
(XLS)

S14 Table Oligonucleotides used in this study. (XLS)
