# Supplementary material for: Intercellular communication is required for trap formation in the nematode-trapping fungus Duddingtonia flagrans
Source: PLoS Genet. 2019 Mar 27;15(3):e1008029. doi: 10.1371/journal.pgen.1008029 (PMC6453484; doi:10.1371/journal.pgen.1008029)
Supplement: S1 Text — (DOCX) [file pgen.1008029.s001.docx]

**S1 Text: Additional genome features**

***D. flagrans g*enome features and phylogenomic tree**

The genome of *D. flagrans* was sequenced with 102 X coverage. The N 50 contig length is 6,168,687 bp. Structural genome annotation estimated 9,927 genes and 10,346 CDS. The average gene density was one gene per 3.8 kb, which is a slightly higher than that of *A. oligospora* (3.50 kb) [1], with an average gene length of 1.67 kb. The average exon length is 485.6 bp with the longest of 8,976 bp. Compared to *A. oligospora*, genes in *D. flagrans* have similar intron repartition (2.75 VS. 2.8) with a slightly larger average intron length (90 VS. 92). Besides the 9,927 genes predicted, 195 tRNAs and 323 rRNAs were predicted by tRNAscan-SE 1.3.1 [2] and the Infernal package 1.1. [3], respectively. The genome of *D. flagrans* consists of 2.18 % of repetitive sequences, which is higher than in *A. oligospora* (0.47 %) [1] and similar to *N. crassa* [4]. *N. crassa* is the first model organism with repeat-induced point (RIP) mutations, referred to as CpG to TpA mutations in duplicated sequences, used as a defense mechanism to suppress the activity of transposases in fungal genomes. We found two proteins in *D. flagrans* with (37.30 % and 39.25 % identity and e-values of E -10 and E -55) to proteins involved in RIP in *N. crassa* (data not show). The presence of these proteins may suggest the presence of the RIP mechanism in *D. flagrans* similar to *A. oligospora* and *Da. haptotyla* [1, 5]. Repetitive sequences in the genome assembly were identified by searching the Repbase database [6] using RepeatMasker and by de novo repetitive sequence search using RepeatModeler (<http://www.repeatmasker.org/RepeatModeler.html>).

We built a phylogenomic tree based on *D. flagrans* genome-encoded orthologous proteins found in the genomes of 13 other filamentous fungi (**suppl. Fig. 2**). The overall phylogeny showed a common ancestor of *D. flagrans* and *A. oligospora* and is in line with recent phylogenetic studies of both species [7].

***D. flagrans* protein annotation**

For a detailed functional annotation, we used the Sma3s tool.[PMC4131829, pmic.201700071]. A total of 6,878 proteins were categorized into functional groups according to Gene Ontology (GO) and pathways (UniProt) (suppl. Fig. S1). 55 % were assigned with a gene name, 52 % with descriptions, 26 % are enzymes, 64 % with GO term, 62 % have keyword and 6 % are involved in pathways. The following GO terms were assigned: ca. 22 % were associated with primary metabolism and less than 0.1 % with secondary metabolism, 4.4 % with transport, 9.5 % with biosynthetic processes and 5.7 % with stress response. Within the molecular function GO domain, we founded 20 % of annotated genes encoded proteins related to hydrolases, lyases, ligases and peptidases while further 10% were related to ion binding. And a total of 664 genes belonging to 61 pathways.  Additionally, *D. flagrans* predicted proteins were annotated using InterProScan. Pfam and IPR domains were assigned to 6,370 and 6,196 proteins, respectively (**suppl. Table S7**). The Pfam domains identified within the *D. flagrans* proteome were compared with those present in the three most closely related fungi **(suppl. Table S8)**. The 4 NTF share high abundance of protein kinases (PF00069), major facilitator superfamily (MFS) transporters (PF00153), mitochondrial carrier proteins, zinc cluster domains (PF00172) and ABC transporters (PF00005). Notably WD domains, G-beta repeats (PF00400) and Ankyrin repeats (3 copies) (PF12796) are the most abundant. Additionally, we found a considerable number of proteins in several protein families related to fungal pathogenicity. For instance, a number of proteases essential for infection as subtilase (**suppl. Table S7**). Other enzyme families such as cellulases, pectinesterases and cellobiohydrolases were detected.

To identify genes involved in pathogenicity in the *D. flagrans* genome, we ran a Blast search against the Pathogen–Host Interaction (PHI) database (phi-base.org, [8] (**suppl. Table S9**) and identified 2,054 proteins in *D. flagrans* (**suppl. Table S9**) similar to *A. oligospora* (2,217). The most abundant PHI proteins encoded transporters and proteins involved in signaling, oxidation, transcription regulation and metabolism (**suppl. Table S10**) as described before for *A. oligospora* and *Da. haptotyla* [5].

**Comparison of putative effector proteins in different NTF**

In the same way as described for *D. flagrans*, the secretomes of 21 other fungi were predicted **(Suppl. Fig. S4 A and suppl. Table S12)**. With few exceptions the secretomes of most fungi represent between 6 and 8 % of the whole genome. A close comparison between nematode trapping fungi **(suppl. Table S12)** showed that *Da. haptotyla* has the largest secretome with about 10 % of the whole genome. Comparing additional fungi, including pathogens and saprophytes, we found that most secretomes represented around 6% of the proteome. Just few proteomes with secretomes representing more than 8% (3 of them nematophagous fungi) were found and fewer presented less than 5 % (mostly human pathogens) (**Suppl. Fig. S4 B**). A Venn diagram was generated between *D. flagrans*, *A. oligospora* and *D. haptotyla* (**Suppl. Fig. S6 A**). *D. flagrans* and *A. oligospora* shared 335 orthologous proteins in the secretome, compared to *A. oligospora* and *D. haptotyla* with less than 200. Reciprocally, 172 proteins were shared between *D. flagrans* and *Da. haptotyla*. The total number of proteins shared between the three fungi was 157. The proteins annotated were enzymes like glycosyl hydrolases, fungal cellulose binding domain, pectate lyase etc. The non-annotated proteins are subject for future research.

The OrthoVenn tool was used to find the best matching cluster of orthologous genes of the four NTF, *D. flagrans*, *A. oligospora*, *Da. haptotyla* and *D. stenobrocha.* The *D. flagrans* secretome comprised of 638 genes that were clustered into 528 clusters of which 139 were shared with the secretomes of the nematode-trapping fungi *A. oligospora*, *Da. haptotyla* and *D. stenobrocha* **(Suppl. Fig. S6 B).** The highest number of clusters was shared between *D. flagrans*, *A. oligospora* and *D. haptotyla*, hence these fungi are closer to each other than to *D. stenobrocha*. Furthermore, 89 proteins of *D. flagrans* had no orthologous proteins with the other three species referred to as singletons.

**References**

1. Yang J, Wang L, Ji X, Feng Y, Li X, Zou C, et al. Genomic and proteomic analyses of the fungus *Arthrobotrys oligospora* provide insights into nematode-trap formation. PLoS Pathog. 2011;7(9):e1002179. Epub 2011/09/13. doi: 10.1371/journal.ppat.1002179. PubMed PMID: 21909256; PubMed Central PMCID: PMCPMC3164635.

2. Lowe TM, Eddy SR. tRNAscan-SE: a program for improved detection of transfer RNA genes in genomic sequence. Nucleic Acids Res. 1997;25(5):955-64. Epub 1997/03/01. PubMed PMID: 9023104; PubMed Central PMCID: PMCPMC146525.

3. Nawrocki EP, Eddy SR. Query-dependent banding (QDB) for faster RNA similarity searches. PLoS Comput Biol. 2007;3(3):e56. Epub 2007/04/03. doi: 10.1371/journal.pcbi.0030056. PubMed PMID: 17397253; PubMed Central PMCID: PMCPMC1847999.

4. Galagan JE, Calvo SE, Borkovich KA, Selker EU, Read ND, Jaffe D, et al. The genome sequence of the filamentous fungus *Neurospora crassa*. Nature. 2003;422(6934):859-68. Epub 2003/04/25. doi: 10.1038/nature01554. PubMed PMID: 12712197.

5. Meerupati T, Andersson KM, Friman E, Kumar D, Tunlid A, Ahren D. Genomic mechanisms accounting for the adaptation to parasitism in nematode-trapping fungi. PLoS Genet. 2013;9(11):e1003909. Epub 2013/11/19. doi: 10.1371/journal.pgen.1003909. PubMed PMID: 24244185; PubMed Central PMCID: PMCPMC3828140.

6. Jurka J, Kapitonov VV, Pavlicek A, Klonowski P, Kohany O, Walichiewicz J. Repbase Update, a database of eukaryotic repetitive elements. Cytogenet Genome Res. 2005;110(1-4):462-7. Epub 2005/08/12. doi: 10.1159/000084979. PubMed PMID: 16093699.

7. Baral H-O, Weber E, Gams W, Hagedorn G, Liu B, Liu X, et al. Generic names in the Orbiliaceae (Orbiliomycetes) and recommendations on which names should be protected or suppressed. Mycological Progress. 2018;17(1-2):5-31.

8. Winnenburg R, Urban M, Beacham A, Baldwin TK, Holland S, Lindeberg M, et al. PHI-base update: additions to the pathogen host interaction database. Nucleic Acids Res. 2008;36(Database issue):D572-6. Epub 2007/10/19. doi: 10.1093/nar/gkm858. PubMed PMID: 17942425; PubMed Central PMCID: PMCPMC2238852.

9. Sievers F, Wilm A, Dineen D, Gibson TJ, Karplus K, Li W, et al. Fast, scalable generation of high-quality protein multiple sequence alignments using Clustal Omega. Mol Syst Biol. 2011;7:539. Epub 2011/10/13. doi: 10.1038/msb.2011.75. PubMed PMID: 21988835; PubMed Central PMCID: PMCPMC3261699.

10. Letunic I, Doerks T, Bork P. SMART: recent updates, new developments and status in 2015. Nucleic Acids Res. 2015;43(Database issue):D257-60. Epub 2014/10/11. doi: 10.1093/nar/gku949. PubMed PMID: 25300481; PubMed Central PMCID: PMCPMC4384020.

11. Daskalov A, Heller J, Herzog S, Fleissner A, Glass NL. Molecular mechanisms regulating cell fusion and heterokaryon formation in filamentous fungi. Microbiol Spectr. 2017;5(2). Epub 2017/03/04. doi: 10.1128/microbiolspec.FUNK-0015-2016. PubMed PMID: 28256191.
