# Supplementary material for: Intercellular communication is required for trap formation in the nematode-trapping fungus Duddingtonia flagrans
Source: PLoS Genet. 2019 Mar 27;15(3):e1008029. doi: 10.1371/journal.pgen.1008029 (PMC6453484; doi:10.1371/journal.pgen.1008029)
Supplement: S1 Fig — Circular map displaying genomic features of the D. flagrans genome. Distinct contigs are depicted using Circos with colored sectors on the outer layer resulting in 36.6 Mb. From outside to inside: (a) CDS position, (b) Intron position, (c) percentage of G+C, (d) percentage of GC skew, (e) effector genes, (f) secretome genes, (g) SSP genes. Inner part: non-coding RNA genes, afu (small nucleolar RNAs known in Aspergillus fumigatus), rRNAs (black) and small nuclear RNAs (snRNAs, green). The original contig numbers provided by Pacific Biosciences were kept in order to match them with the published sequence. (PPTX) [file pgen.1008029.s002.pptx]

## Slide 1
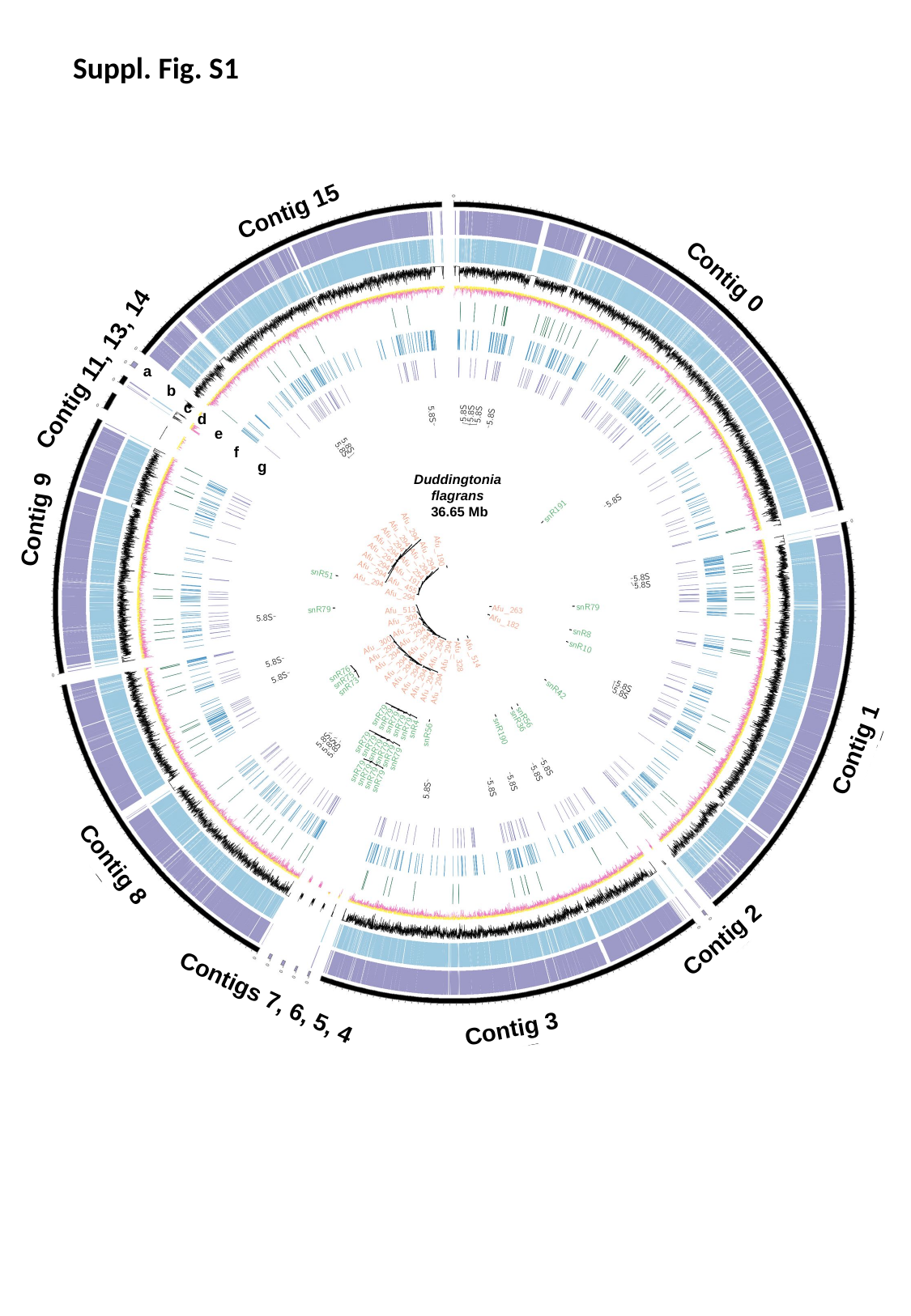

Suppl. Fig. S1
Contig 15
Contig 0
Contig 11, 13, 14
a
b
c
d
e
f
g
Duddingtonia
flagrans
36.65 Mb
Contig 9
Contig 1
Contig 8
Contig 2
Contigs 7, 6, 5, 4
Contig 3
