# Supplementary figures and images for: Intercellular communication is required for trap formation in the nematode-trapping fungus Duddingtonia flagrans
Source: PLoS Genet. 2019 Mar 27;15(3):e1008029. doi: 10.1371/journal.pgen.1008029 (PMC6453484; doi:10.1371/journal.pgen.1008029)

## Slide 1
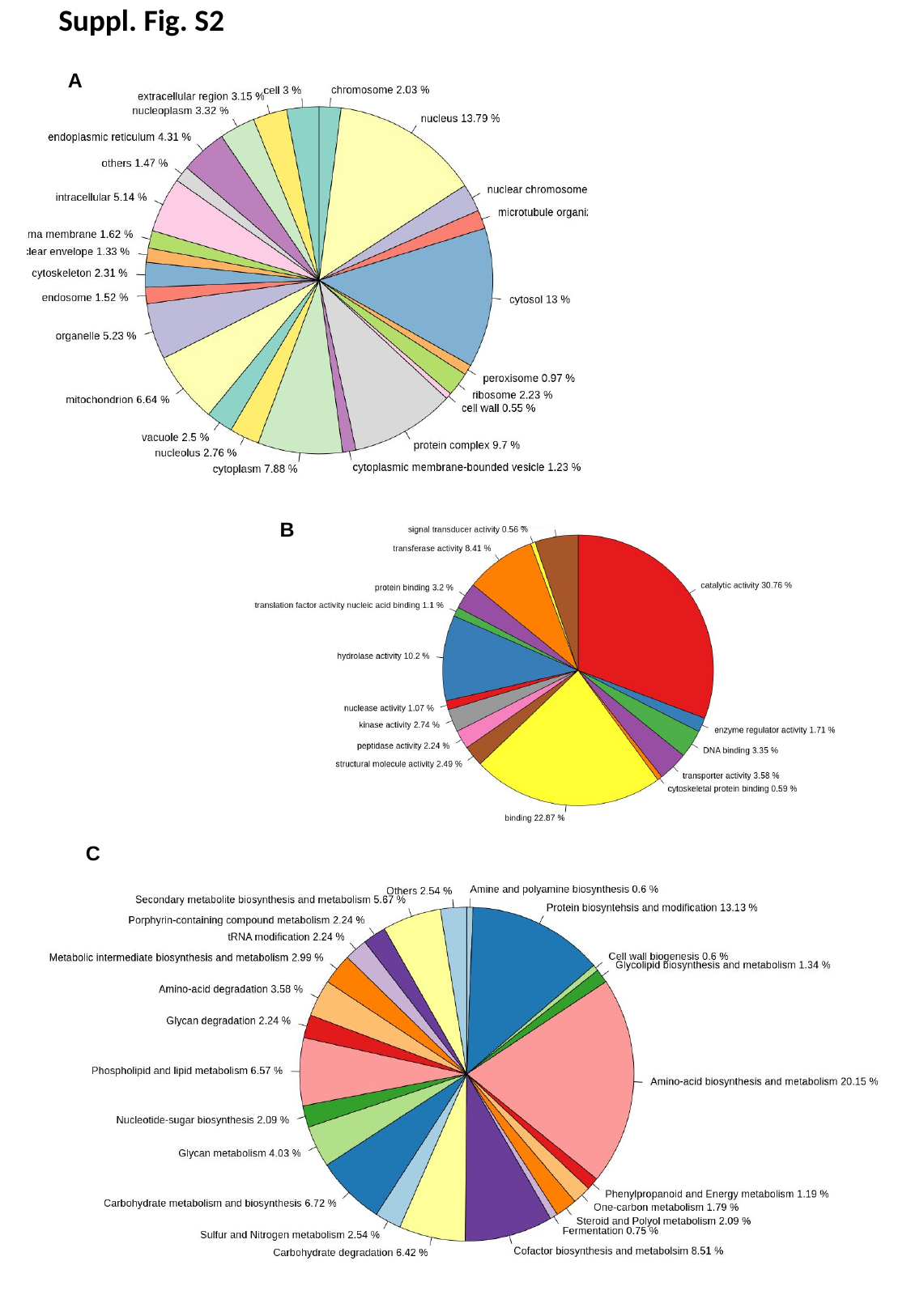

Suppl. Fig. S2
A
B
C

Supplement: S2 Fig — The slices represent the percentage of unigenes identified in the particular category. (A) Biological process. (B) Molecular function. (C) Uniprot pathways. (PPTX) [file pgen.1008029.s003.pptx]

## Slide 1
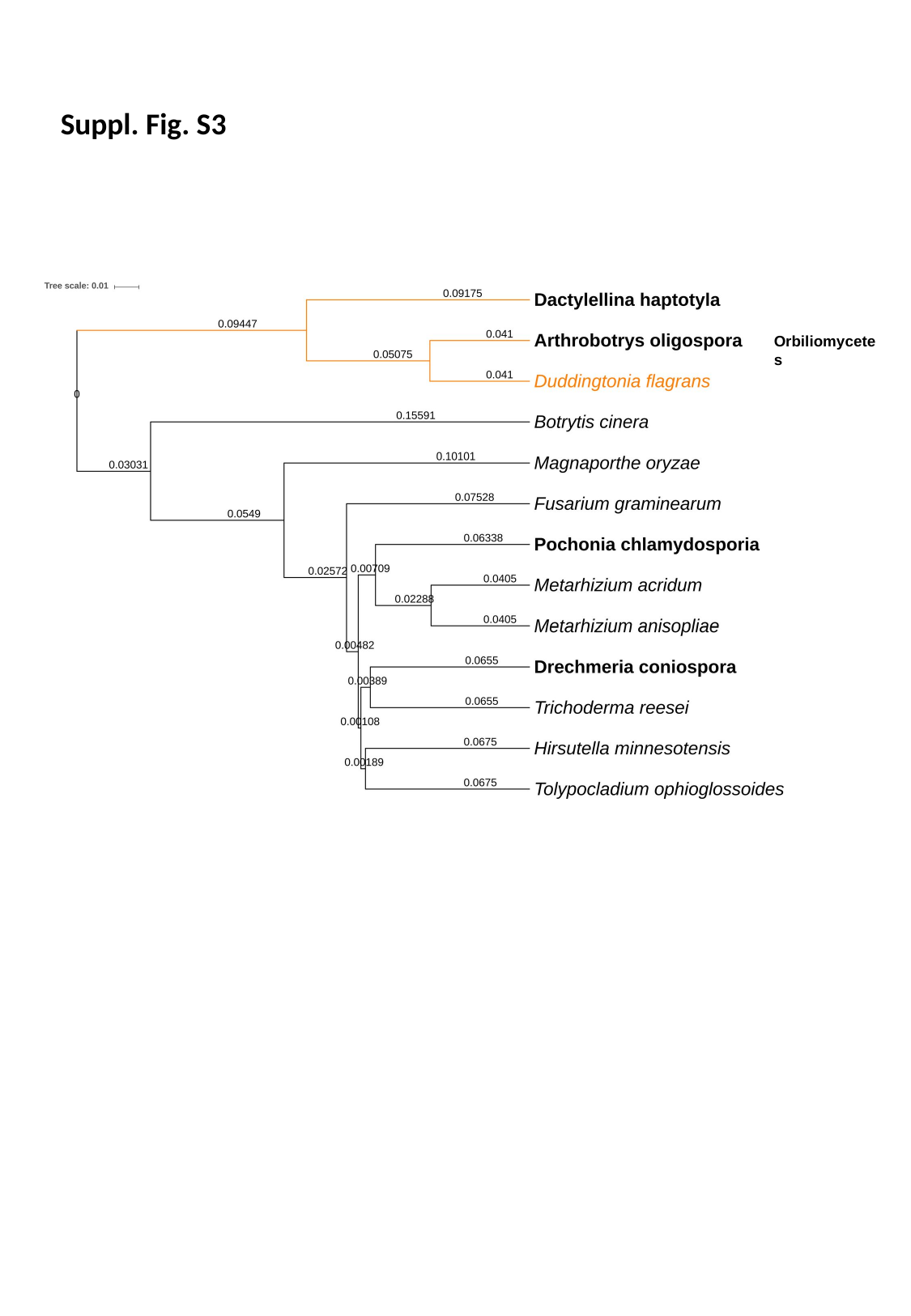

Suppl. Fig. S3
Orbiliomycetes

Supplement: S3 Fig — Bootstrap values are indicated beside the nodes. Orange color indicates the clade grouping of nematode trapping fungi. The proteomes of Arthrobotrys oligospora (ADOT00000000.1), Drechmeria coniospora (LAYC00000000.1), Dactylellina haptotyla (AQGS00000000.1), Fusarium graminearum (AACM00000000.2), Hirsutella minnesotensis (JPUM00000000.1), Metarhizium acridum (ADNI00000000.1), Metarhizium anisopliae (AZNF00000000.1), Magnaporthe oryzae (AZNF00000000.1), Pochonia chlamydosporia (LSBJ00000000.2), Tolypocladium ophioglossoides (LFRF00000000.1) and Trichoderma reesei (AAIL00000000.2) were downloaded from NCBI and compared using BLASTP against the D. flagrans proteome. The matches with at least E value < = 1E-100 and at least 70% sequence identity over 85% of the protein lengths were taken as homologous sequences. The set of 93 high-confidence orthologous proteins were concatenated and aligned using Clustal Omega [76] and phylogenetic distances were calculated using the maximum likelihood-based method implemented within MEGA using default parameters. Tree Of Life (iTOL) v3 was used to visualize MEGA output [77]. (PPTX) [file pgen.1008029.s004.pptx]

## Slide 1
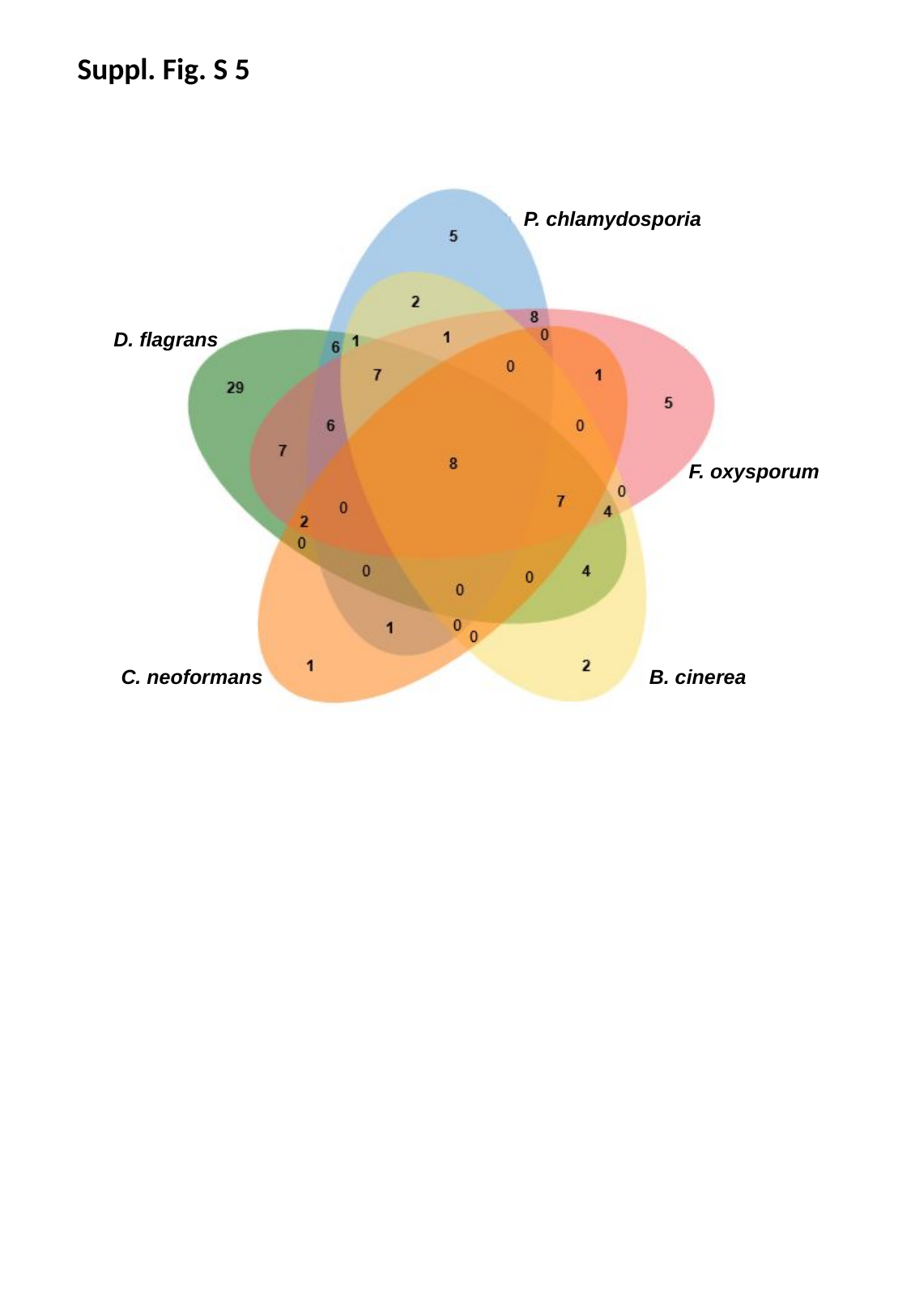

Suppl. Fig. S 5
P. chlamydosporia
D. flagrans
F. oxysporum
C. neoformans
B. cinerea
C. albicans

Supplement: S5 Fig — The OrthoVenn diagram shows the clustering of orthologous proteins exclusive to D. flagrans (not found in A. oligospora) in the five chlamydospore forming fungi Botrytis cinerea, Cryptococcus neoformans, Fusarium oxysporum and Pochonia chlamydosporia. The comparison of the proteomes of D. flagrans and A. oligospora resulted in 591 proteins exclusive to D. flagrans that could represent candidates involved in chlamydospore 46 formation. These proteins were compared to the proteomes of five chlamydospore forming fungi and then clustered using OrthoVenn. The 591 D. flagrans proteins formed 81 clusters including 8 clusters that can be found in five chlamydospore forming fungi. (PPTX) [file pgen.1008029.s006.pptx]

## Slide 1
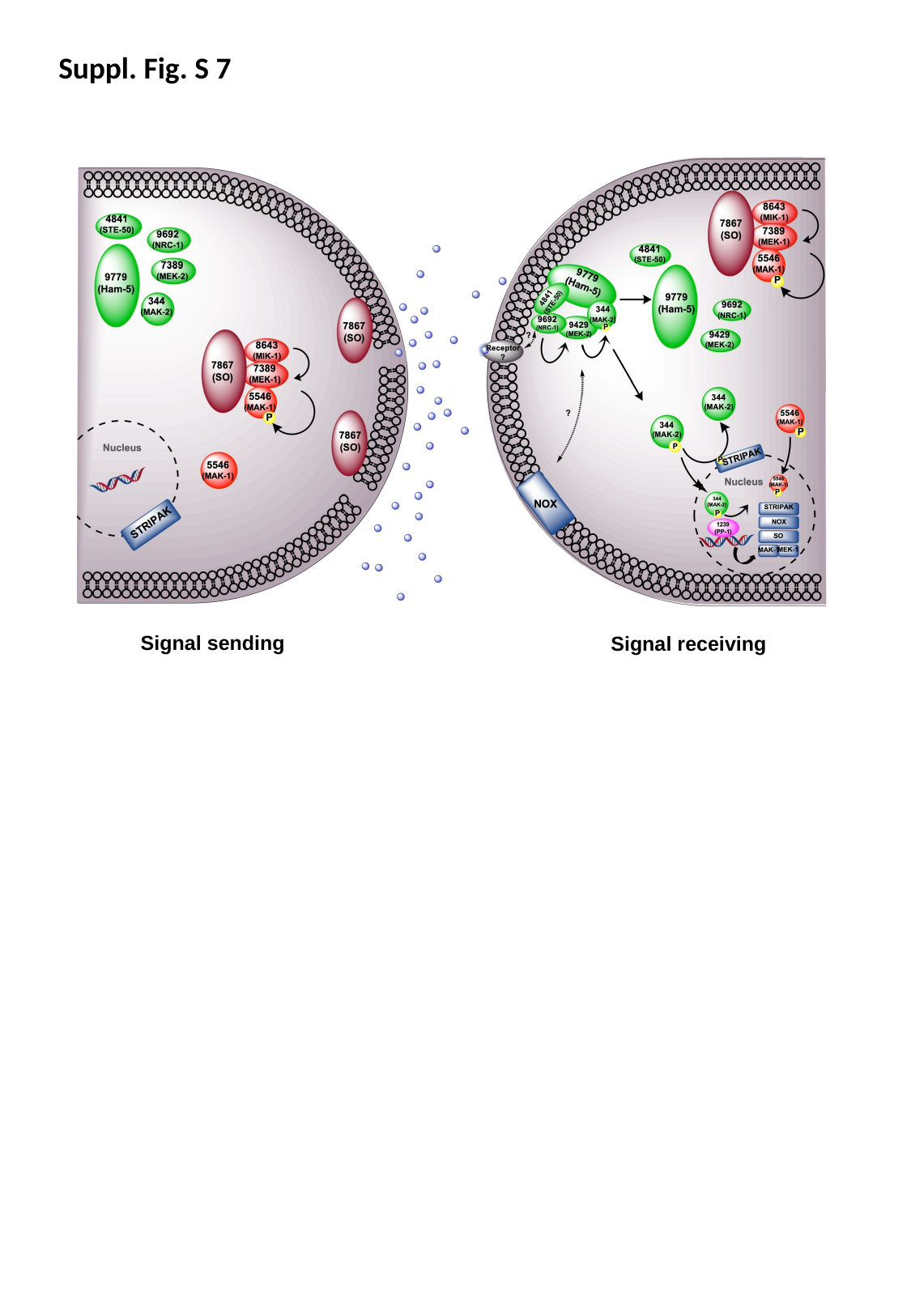

Suppl. Fig. S 7
Signal sending
Signal receiving

Supplement: S7 Fig — The pathway was reconstructed using the N. crassa model [52]. A so far unknown signaling molecule is released by the signal-emitting cell, probably by exocytosis, in a SO-dependent manner. The signal is perceived by a so far unknown receptor of the signal-receiving cell. The binding leads to the assembly and activation of the MAK-2 module at the plasma membrane, which causes phosphorylation of MOB-3 of the 47 STRIPAK complex, and subsequent entry of MAK-1 into the nucleus. Inside the nucleus gene expression of cell fusion-relevant genes is controlled by the MAK-2 activated transcription factor PP-1. The activation of MAK-2 involves the production of reactive oxygen species by the NADPH oxidase (NOX) complex. MAK-2 is assembled at the plasma membrane with the scaffold protein HAM-5, the adaptor protein STE50 and its two upstream kinases MEK-2 and NRC-1 in an oscillating manner. The D. flagrans orthologues are displayed as numbers without the DFL_ prefix. (PPTX) [file pgen.1008029.s008.pptx]
