# Supplementary material for: Intercellular communication is required for trap formation in the nematode-trapping fungus Duddingtonia flagrans
Source: PLoS Genet. 2019 Mar 27;15(3):e1008029. doi: 10.1371/journal.pgen.1008029 (PMC6453484; doi:10.1371/journal.pgen.1008029)
Supplement: S4 Fig — 21 fungal secretomes and effectors were predicted and were compared to those of D. flagrans. All secretomes were generated on the same way as for D. flagrans (See Materials and methods). The proteomes of A. oligospora (ADOT00000000.1), Fusarium graminearium (AACM00000000.2), Aspergillus nidulans (AACD00000000.1), Dre. coniospora (LAYC00000000.1), D. stenobrocha (ASQI00000000.1), Ustilago maydis (AACP00000000.2), Neurospora crassa (AABX00000000.3), Dactylellina haptotyla (AQGS00000000.1), Penicillium brasilianum (CDHK00000000.1), Aspergillus fumigatus (AAHF00000000.1), Candida albicans (GCA_000182965.3), Candida galabrata (GCF_000002545.3), Alternaria alternata (LXPP00000000.1), Magnaporthe oryzae (AACU00000000.3), Trichoderma reesei (AAIL00000000.2), Pochonia chlamydosporia (LSBJ00000000.2), Botrytis cinera (GCA_000143535.4), Metarhizium acridum (ADNI00000000.1), Metarhizium album (AZHE00000000.1), Hirsutella minnesotensis (JPUM00000000.1), Cordyceps militaris (AEVU00000000.1) were downloaded from NCBI. Venn diagrams were generated using the VennDiagram package in R. (A) The secretomes and putative effectors of 22 fungi are compared. The purple color presents secretome proteins frequency and grey color presents effectors protein frequency. (B) Comparison of nematophagous, insect pathogens, human pathogens, plant pathogens and saprophytic fungi proteome sizes relative to the secretome sizes. Lines in grey, red and blue represent limits of 1%, 5% or 8% of secreted proteins respectively. (PPTX) [file pgen.1008029.s005.pptx]

## Slide 1
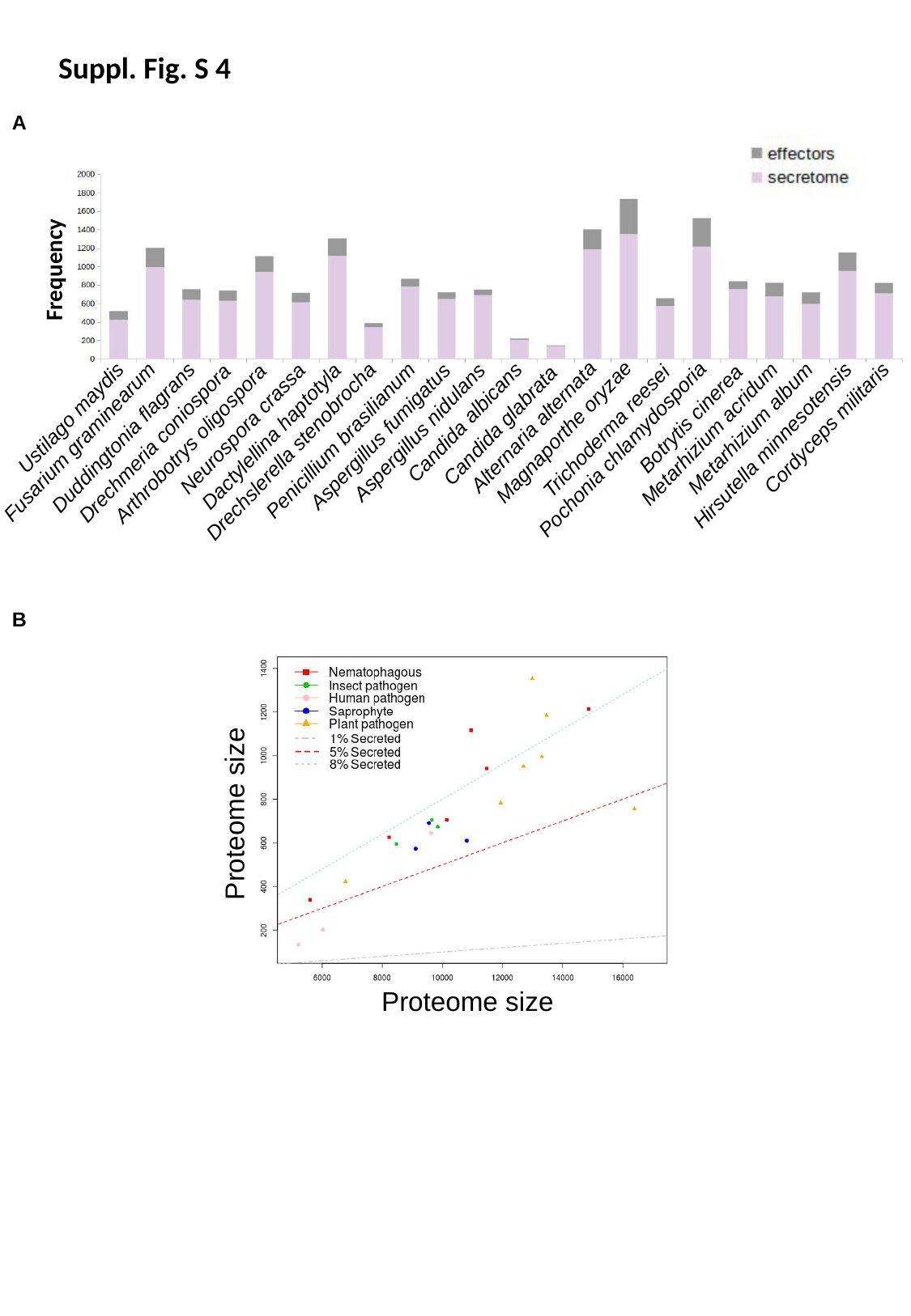

Suppl. Fig. S 4
A
Frequency
Ustilago maydis
Botrytis cinerea
Candida albicans
Candida glabrata
Alternaria alternata
Cordyceps militaris
Metarhizium album
Neurospora crassa
Trichoderma reesei
Magnaporthe oryzae
Aspergillus nidulans
Metarhizium acridum
Dactylellina haptotyla
Aspergillus fumigatus
Duddingtonia flagrans
Penicillium brasilianum
Fusarium graminearum
Drechmeria coniospora
Arthrobotrys oligospora
Hirsutella minnesotensis
Pochonia chlamydosporia
Drechslerella stenobrocha
B
Proteome size
Proteome size
