# Supplementary material for: Intercellular communication is required for trap formation in the nematode-trapping fungus Duddingtonia flagrans
Source: PLoS Genet. 2019 Mar 27;15(3):e1008029. doi: 10.1371/journal.pgen.1008029 (PMC6453484; doi:10.1371/journal.pgen.1008029)
Supplement: S6 Fig — (A) Venn diagram of D. flagrans, A. oligospora and D. haptotyla secretomes, 157 orthologous proteins are shared. (B) Venn diagram of orthologous gene clusters of D. flagrans, A. oligospora, Da. haptotyla and D. stenobrocha. The four nematode trapping fungi share 139 clusters. (PPTX) [file pgen.1008029.s007.pptx]

## Slide 1
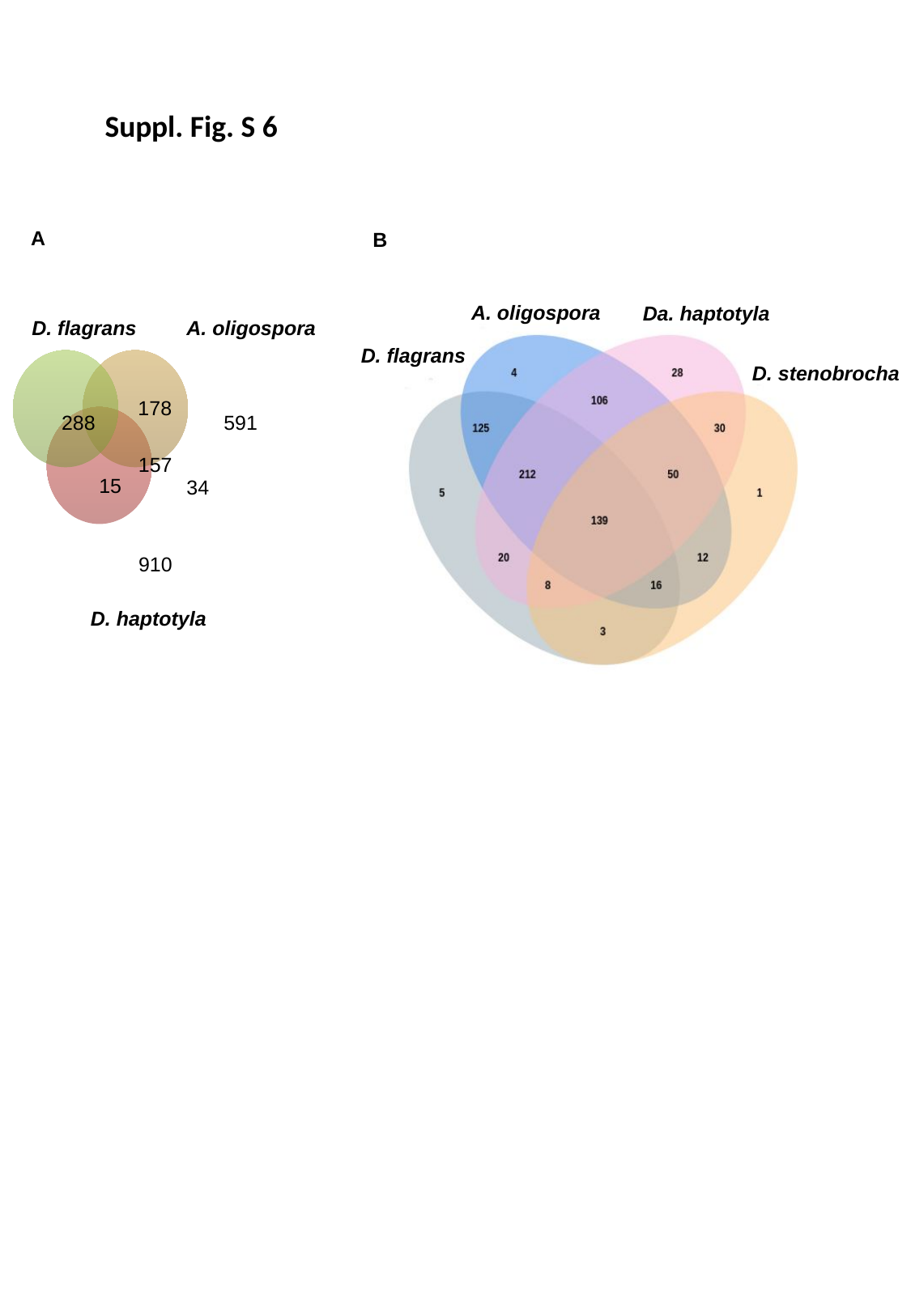

Suppl. Fig. S 6
A
B
A. oligospora
Da. haptotyla
D. flagrans
D. stenobrocha
D. flagrans
A. oligospora
178
288
591
157
15
34
910
D. haptotyla
